# Design and Characterization of Novel Gastroretentive Drug Delivery System of Antibiotics and Piperine for the Eradication of *H. pylori* Infection

**DOI:** 10.1021/acs.molpharmaceut.5c01253

**Published:** 2025-11-14

**Authors:** Ashutosh Gupta, Moumita Saha, Shivani Shailesh Kunkalienkar, Aadarsh Ghurye, Shweta Verma, Jahnavy Joshi, Abhishek Jha, Srinivas Mutalik, Shiran Shetty, Raghu Chandrashekar Hariharapura, Ashwini Aithal, K Nandakumar, Raviraja N. Seetharam, Sudheer Moorkoth

**Affiliations:** † Department of Pharmaceutical Quality Assurance, Manipal College of Pharmaceutical Sciences, 76793 Manipal Academy of Higher Education, Manipal, Karnataka 576104, India; ‡ Department of Pharmaceutical Biotechnology, Manipal College of Pharmaceutical Sciences, 76808 Manipal Academy of Higher Education, Manipal, Karnataka 576104, India; § Manipal Centre for Biotherapeutics Research, 76793 Manipal Academy of Higher Education, Manipal, Karnataka 576104, India; ∥ Department of Pharmaceutics, 79203 Dr. D. Y. Patil Institute of Pharmaceutical Sciences and Research Pimpri, Pune 411018, India; ⊥ Department of Pharmaceutics, Manipal College of Pharmaceutical Sciences, 76808 Manipal Academy of Higher Education, Manipal, Karnataka 576104, India; # Department of Gastroenterology and Hepatology, Kasturba Medical College, 29224 Manipal Academy of Higher Education, Manipal, Karnataka 576104, India; ∇ Division of Anatomy, Department of Basic Medical Sciences, 76793 Manipal Academy of Higher Education, Manipal, Karnataka 576104, India; ○ Department of Pharmacology, Manipal College of Pharmaceutical Sciences, 76808 Manipal Academy of Higher Education, Manipal, Karnataka 576104, India

**Keywords:** *Helicobacter pylori*, gastroretentive drug delivery system, gastric ulcer, antibiotics, antibacterial effect

## Abstract

*Helicobacter pylori* (*H. pylori*) infection affects about half the world population, and if left untreated, can cause painful sores in the stomach lining and intestinal bleeding, leading to peptic ulcer disease (PUD) and stomach cancer. Treatment of *H. pylori* infection is always a challenge to the treating doctor because of the treatment inefficiency resulting from the poor bioavailability of the drug at the inner layers of the gastric mucosa, where the bacteria reside. This also results in the development of antibiotic resistance. In this work, we developed a mucoadhesive gastroretentive drug delivery system (M-GRDDS) for the effective delivery of antibiotics and piperine to the gastric mucosa. The GRDDS system was formulated by using the ion-gelation method and was evaluated for its entrapment efficiency, particle size, swelling behavior, drug release, mucoadhesion property, and *H. pylori* eradication efficacy. The efficacy of the drug-loaded mucoadhesive GRDDS formulation was compared with that of the free drug. Results showed that the percentage entrapment efficiency was more than 80% for all the drugs. M-GRDDS beads showed controlled release at pH 1.2 and 7.4. The optimized mucoadhesive beads showed good *in vitro* mucoadhesion in X-ray photography, with a mean gastric residence time of more than 8 h in rabbits. Tissue distribution study in rats revealed local delivery of the drugs to the gastric mucosa from the M-GRDDS beads. The *in vivo* efficacy study performed on Sprague–Dawley rats showed that the colony-forming units in the group treated with the novel GRDDS formulation were fewer than those in the group treated with the free drugs. Biochemical tests, gene expression studies, and histopathology studies corroborated the enhanced efficacy of the M-GRDDS formulation in eradicating the infection and curing peptic ulcers. The results conclude that the developed M-GRDDS formulation holds significant potential for improving local bioavailability, contributing to the more effective eradication of *H. pylori*-based gastric ulcers.

## Graphical abstract

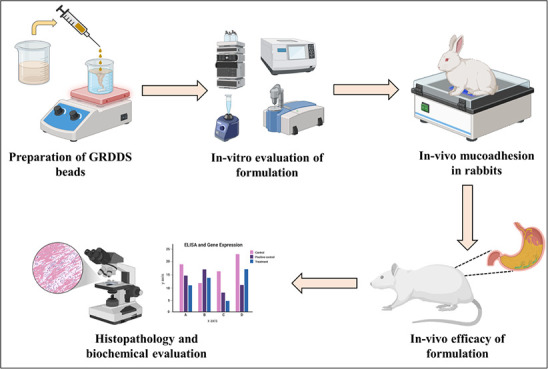

## 1 Introduction

*Helicobacter pylori* (*H. pylori*), commonly known as *H. pylori*, is a helical rod-shaped, Gram-negative bacterium. It requires only low oxygen levels to survive and colonize in the stomach’s mucosa. If left untreated, *H. pylori* infection may lead to gastritis, peptic ulcer, gastric cancer, and mucosa-associated lymphoid tissue lymphoma (MALT).^1–3^ Currently, more than 50% of the global population is estimated to be infected with this bacterium.^4,5^ *H. pylori* is also associated with extra-intestinal manifestations, including short stature, refractory iron deficiency anemia (IDA), immune thrombocytopenic purpura, Alzheimer’s disease, vitamin B_12_ deficiency and psychiatric disorders.^6^ The bacterial pathogenesis occurs primarily via adaptation of *H. pylori* in the gastric acidic environment by the urease enzyme that hydrolyzes urea to ammonia, which is further used for neutralizing stomach acid.^4,7,8^ In the gastric fluid, the bacterial flagella allow rapid movement of bacteria from the lumen to the mucosa, producing adhesins to colonize on the surface of gastric epithelium.^9^ After attaching to the gastric epithelium, the bacteria release toxins that ensure the bacteria’s survival by disrupting the signaling pathway in the host cells and inhibiting T-cell proliferation.^10^ Specifically, VacA and CagA endotoxins released by *H. pylori* act by disrupting cell structure integrity of the gastric epithelium cells and lead to secretion of inflammatory cytokines, which in turn results in cell apoptosis.^11^ This leads to damage of the gastric epithelial cells, allowing the hydrochloric acid to sip in that area and further damaging the surrounding cells, resulting in stomach ulcers. More than 90% of the population of *H. pylori* is found to be extracellular. However, a very small population has been found to reside in intracellular vacuoles, where it can escape the antibiotics and come to the extracellular space to repopulate the bacteria during favorable conditions.^12^

Treatment of *H. pylori* infection is always a challenge and a significant concern for the treating doctor because of the ineffectiveness of the currently available treatment regimens to completely cure the infection resulting in antibiotic resistance. Presently approved treatment for combating *H. pylori* is a combination of two or more antibiotics in the traditional delivery mode, which fails in the eradication of *H. pylori* infection because of the insufficient reach of antibiotics and poor bioavailability to the inner mucosal layer of the stomach lining where the bacteria reside.^13–22^ These often results in treatment failure due to antibiotic resistance, poor patient compliance due to polypharmacy, and leads to omission of dosage due to the complexity of multiple medications (6–12 pills/day).^23–25^ Since the medication does not reach the intended site of action, many antibiotics exhibit poor *in vivo* effectiveness. The medication needs to be readministered at predetermined intervals to maintain a sufficient dose.

The best way to enhance drug bioavailability and boosting medication’s effectiveness in this scenario is to use controlled drug delivery. Among the several methods of guided delivery, the gastro-retentive drug delivery system (GRDDS), allows the release of medication sustainably and holds the formulation in the stomach for a longer time. GRDDS also exhibits localized action in the stomach mucosa by supplying a significant quantity of medication at the site of action.^26–28^ Higher antibiotic concentrations in the stomach area where *H. pylori* reside can be maintained by prolonged drug retention, which enhances treatment effectiveness. Among the many GRDDS approaches such as floating, mucoadhesion (bioadhesive), high density and expandable systems, the mucoadhesive system can be advantageous as the *H. pylori* bacteria resides in the gastric mucosa.

Based on this hypothesis, we prepared and evaluated chitosan-coated sodium alginate mucoadhesive-GRDDS (M-GRDDS) beads of amoxicillin trihydrate (AMO), metronidazole (MTZ), and piperine (PIP) for their *H. pylori* eradication potential in animal models. AMO and MTZ were chosen since they were used in the first line of treatment. AMO exhibits bactericidal properties by inhibiting cell wall biosynthesis. Using MTZ also has an advantage in acting on the dormant intracellular anaerobic adaptation of *H. pylori*.^29^ Piperine, a P-gp inhibitor, was included in the treatment regimen as an adjuvant considering its anti-inflammatory, antioxidant, and antiulcer properties, which may help in ulcer.^30–32^ Co-administration of PIP with AMO has also been reported to enhance the bioavailability of AMO.^33^ Some studies have shown that PIP can reduce gastric inflammation and oxidative stress and inhibit the growth of *H. pylori*.^31^ PIP has also been shown to increase gastric mucus production, which could help protect the stomach lining from damage.^34^ PIP inhibits *H. pylori* motility and adhesion to gastric cells by downregulating key flagellar genes, particularly *flhA* and *flgE*.^31^ It also inhibits the translocation of *H. pylori* toxins, such as CagA and VacA, into host cells.^35^ This suppression reduces bacterial movement toward the gastric epithelium, thereby minimizing colonization and virulence factor injection.^36^ Pantoprazole (PAN), an acid-suppressing agent, is also part of the first-line treatment. Enteric-coated PAN beads were also prepared using Eudragit L30 D-55 polymer since PAN is unstable in the acidic environment of the stomach.

## 2 Materials and Methods

### 2.1 Materials

Amoxicillin trihydrate (off-white powder; purity >98%) was obtained as a gift sample from Sun Pharma, Gurgaon, Haryana, India. Metronidazole (white crystalline powder; purity >98%) procured from the Combi Block, USA. Pantoprazole sodium was obtained as a gift sample from Sun Pharma, Gurgaon, Haryana, India. Piperine was procured from Sigma-Aldrich, Bengaluru, India. Hydrogen peroxide, (30%) and sodium hydroxide pellets (purity ≥98%) were procured from Himedia Laboratories Pvt. Ltd., Mumbai, India. Orthophosphoric acid (88%) was procured from Merck Ltd., Mumbai, India. Methanol (MeOH) and acetonitrile (ACN) used for HPLC method development were of HPLC grade and obtained from Finar Ltd., Ahmedabad, India. Purified water (Milli-Q) was generated in-house using Direct-Q 3 water purification system, Millipore Corporation, Billerica, USA. Hydrochloric acid (35% pure AR), was procured from Finar Ltd., Ahmedabad, India. Membrane filter (0.45 μm) was obtained from Riviera Glass Pvt. Ltd., Mumbai, India. Other reagents, such as potassium dihydrogen phosphate (purity >98%), and sodium hydroxide, were procured from Merck Laboratories Pvt. Ltd., Mumbai, India. HyperClone ODS C18 column (5 μm particle size, 120 Å, 250 mm × 4.6 mm) was procured from Phenomenex, Hyderabad, India. Sodium alginate and chitosan (purity >98%) were procured from Loba Chemie, Mumbai, India. Calcium chloride (purity >98%) was procured from Merck Laboratories Pvt. Ltd. The solvents and reagents used for the method development and validation were HPLC-grade chemicals. ELISA kits prostaglandin E2 (PGE2) (Cat# E-EL-0034) procured from Elabscience, New Delhi, India. Cyclooxygenase-2 (COX-2) (Cat# E-EL-0034) was procured from Antibodies Online, Cambridge, United Kingdom. Tumor necrosis factor-alpha (TNF-α) (Cat# KRC3011) and interleukin-1 beta (IN-1 β) (Cat# BMS630) were procured from Thermo Fisher Scientific, Bengaluru, Karnataka, India. Radioimmunoprecipitation assay (RIPA) buffer was procured from Himedia Laboratories Pvt. Ltd., Mumbai, India. Tri reagent and nuclease-free water were procured from Thermo Fisher Scientific, Bengaluru, Karnataka, India. Isopropyl alcohol was procured from Sigma-Aldrich Bengaluru, India. cDNA kit was procured from DSS Takara Bio India Pvt. Ltd., New Delhi, India. Primers for the gene expression of TNF-α and COX-2 were procured from Sigma-Aldrich Bengaluru, India. Hematoxylin/eosin dye was procured from Molychem, Mumbai, Maharashtra, India.

### 2.2 Preparation of Mucoadhesive-GRDDS (M-GRDDS) Beads of the Proposed Drugs

#### 2.2.1 M-GRDDS Beads of Amoxicillin, Metronidazole, and Piperine

The mucoadhesive-GRDDS beads of the AMO, MTZ, and PIP was prepared separately by the ionic gelation method, employing chitosan and sodium alginate as mucoadhesive polymers.^37^ Sodium alginate was chosen because of its ability to release the drug sustainably.^38^ It also helps in targeted delivery of the drug to the gastric mucosa,^39^ and increased drug bioavailability.^40^ Chitosan is a natural polysaccharide (biopolymer) obtained by alkaline deacetylation of chitin, also is reported to help in controlled drug release.^41^ Initially, a polymeric solution of sodium alginate was prepared in water, and the respective drugs were added. A solution of chitosan was prepared in 1% v/v acetic acid (pH adjusted to 5.0) with continuous stirring at 50 rpm on a magnetic stirrer, and calcium chloride was added to this solution. In the next step, the drug containing sodium alginate solution was added dropwise to the chitosan solution using 18G needle to form the beads. A general flowchart for its preparation is provided in Figure 1. After waiting for 15 min, the prepared beads were removed from the polymeric solution, washed with demineralized water, and dried at 40 °C for 6 h. Different polymer ratios were tried for formulating the beads loaded with the drugs (Supplementary Tables S1–S3). Among these trials, formulations containing 7% calcium chloride, 0.5% chitosan, and 9% sodium alginate demonstrated improved entrapment efficiency of the selected drugs. The prepared beads were stored in desiccators until used.

**Figure 1:**
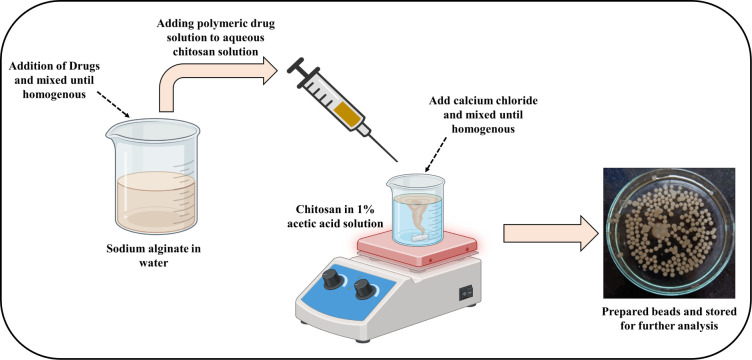
General procedure for the development of the mucoadhesive-GRDDS beads.

#### 2.2.2 Enteric-Coated Beads of Pantoprazole

Eudragit L 30 D-55 polymer was used for the enteric coating. Previously prepared sodium alginate-chitosan beads of PAN were transferred to the Eudragit L 30 D-55 solution in water with continuous stirring at 50 rpm for 15 min. The enteric-coated beads were removed from the polymeric solution, washed with demineralized water, dried at 40 °C for 6 h, and stored in a desiccator. Different trial conditions for preparing enteric-coated PAN beads are presented in supplementary data (Supplementary Table S4).

### 2.3 Characterization of the Prepared Formulations

#### 2.3.1 Appearance and Bead Size

Size of the drug-loaded M-GRDDS beads was assessed by observing them through a microscope. Drug-loaded M-GRDDS beads were arranged on a slide, and the diameters of 40 beads were measured using a calibrated eyepiece and stage micrometer. The mean diameter was calculated using eq 1.

1 $$\mathrm{Average}\;\mathrm{diameter}=\frac{\Sigma nd}{n}\times C.F$$

Where, *n* = number of beads, *d* = diameter of the beads, C.F = calibration factors

#### 2.3.2 % Swelling Measurement

Previously weighed drug loaded M-GRDDS beads were taken separately in beakers containing 50 mL of 0.1 M HCl (pH = 1.2) and phosphate buffer (pH = 7.4) at 37 °C for 10 h in each buffer. At specific intervals throughout the 10-h period, the beads were taken out, dried on paper towels, and weighed.^42^ The % swelling of beads was calculated using eq 2

2 $$\%\mathrm{Swelling}=\frac{\left(Wm-Wt\right)}{Wt}\times 100$$

Where, Wt = initial weight of beads, Wm = denotes the weight at equilibrium of beads

#### 2.3.3 Determination of Drug Entrapment Efficiency of Drug-Loaded M-GRDDS Beads

Weighed 100 mg of the drug loaded M-GRDDS beads of each drug and placed them in a beaker containing 100 mL of 0.1 N HCl. The contents of the beaker were stirred on a magnetic stirrer for 24 h at 37 °C. After 24 h, the solution was filtered through Whatman filter paper (0.45 μm). The polymeric debris was washed twice with a fresh buffer to extract any adhered drug. The filtrate was diluted and analyzed using the previously published RP-HPLC analytical method.^43,44^ The % entrapment efficiency was calculated using eq 3

3 $$\%\mathrm{Entrapment}\;\mathrm{efficiency}=\frac{\mathrm{Estimated}\;\%\;\mathrm{drug}\;\mathrm{content}}{\mathrm{Theoretical}\;\%\;\mathrm{drug}\;\mathrm{content}}\times 100$$

#### 2.3.4 Surface Morphology Studies

The surface morphology of the prepared drug-loaded M-GRDDS beads was evaluated using a scanning electron microscope (SEM) (Gemini SEM 300–820201722). The sample was placed on stubs using a carbon adhesive tape, coated with gold palladium alloy using a fine coat ion sputter, and the surface was analyzed using SEM.^45^

#### 2.3.5 Fourier Transform Infrared Spectroscopy (FTIR) Studies

FTIR study was used to check the interaction between the pure drug and excipients. The FTIR spectra were captured using a Shimadzu IR spectrophotometer (IRAffinity-1S) across the 4000–400 cm^–1^ range. To prepare each sample, it was mixed with KBr, ground using a mortar and pestle, and then compressed into a disc under pressure of around 1000 psi.^46^

#### 2.3.6 X-ray Diffraction (XRD) Analysis

The X-ray diffractometer (Rigaku Co., Tokyo, Japan) was used to capture the XRD patterns of the drug-loaded M-GRDDS beads. It operated at 600 W with a constant voltage of 40 kV and a fixed tube current of 15 mA. A graphite monochromator was used for X-ray diffraction, detected via a standard scintillation counter. The diffraction intensities were recorded within the 5–80° (2θ) range.

#### 2.3.7 Differential Scanning Calorimetry (DSC) Studies

Thermal properties of the prepared drug-loaded M-GRDDS beads were examined utilizing DSC (Shimadzu-TA-60 WS, Kyoto, Nagoya, Japan). This involved loading 5–10 mg of the sample into an aluminum pan, sealing, and subjecting it to temperature from 30 to 200 °C at a rate of 10 °C/min under a nitrogen flow of 40 mL/min. An empty aluminum pan was employed as a reference.^47^

#### 2.3.8 *In Vitro* Floating Study

The in vitro floating study was performed in a USP dissolution test apparatus, Lab India. Twenty beads from each drug-loaded M-GRDDS formulation were placed in a dissolution vessel having 500 mL of 0.1 N HCl (pH 1.2) and phosphate buffer (pH 7.4) controlled at 37 ± 0.5 °C and agitated at 50 rpm. The time took for the beads to sink to the bottom of the vessel after introduction (floating lag time) was recorded using a stop watch.^47^

#### 2.3.9 *In Vitro* Mucoadhesion Study

*In vitro* mucoadhesion of the drug-loaded M-GRDDS beads was evaluated by the wash-off method. Forty beads were evenly placed on a rat stomach mucosa slice measuring 5 cm width and 3 cm length. The slice was set on a Perspex mounting block and incubated for 20 min. The block was then tilted at 30-degree angle while keeping the temperature and humidity of the chamber at 37 ± 1 °C and 90 ± 2% RH, respectively. The tissue was then exposed to 0.1 N HCl (pH 1.2) and phosphate-buffered saline (PBS) (pH 7.4) for 8 h at a steady flow rate of 1 mL/min. The % mucoadhesion was calculated using eq 4

4 $$\%\mathrm{Mucoadhesion}=\frac{\mathrm{No}-\mathrm{Ni}}{\mathrm{No}}\times 100$$

where, No = number of beads applied initially and Ni = number of beads rinsed from the tissue.

#### 2.3.10 *In Vitro* Drug Release Study

Release study was performed on the drug loaded M-GRDDS beads of AMO, MTZ, and PIP equivalent to 100 mg of drug. The study was performed using the USP dissolution apparatus II (Lab India) in 500 mL of 0.1 N HCl (pH 1.2 stomach condition) and phosphate buffer (pH 7.4 intestinal condition) with an agitation speed of 100 rpm at 37 ± 0.5 °C. Release of the enteric-coated beads of PAN was studied at intestinal pH only (pH 7.4 phosphate buffer). Aliquots of 2 mL were withdrawn at predetermined intervals and replaced immediately with the same volume of fresh dissolution medium to maintain the sink conditions. Collected samples were suitably diluted and analyzed by HPLC at their respective wavelength.

#### 2.3.11 *In Vitro* Release Kinetics Study

The release kinetics and fundamental mechanisms controlling drug release from the drug loaded chitosan-coated alginate beads were determined by analyzing and comparing the drug release data using a variety of mathematical models. The models utilized included Higuchi, Korsmeyer-Peppas, zero-order, and first-order models, all of which shed light on the pattern and rate of drug release. A sustained release mechanism has shown zero-order release kinetics, which shows a steady drug release rate throughout time, irrespective of drug concentration. In contrast, the first-order model represents a concentration-dependent drug release, in which the rate falls as the drug is released from the beads. As frequently seen in matrix-based drug delivery systems, the Higuchi model represents drug release as a diffusion-controlled process in which the drug is released proportionately to the square root of time. The mechanism of drug release has been further explained by applying the semiempirical Korsmeyer-Peppas model, where the diffusion exponent (n) serves to determine whether the release follows case II transport (swelling-controlled release), non-Fickian (anomalous) transport, or Fickian diffusion. The best release kinetic model was found by fitting the release data to these models, explaining the drug release pattern from the formulation. Additionally, this research shed light on whether drug release from the M-GRDDS beads was primarily mediated by diffusion, swelling, erosion, or a mixture of these modes.

### 2.4 *In Vivo* Mucoadhesion Study in Rabbits

The *in vivo* mucoadhesion ability was evaluated in rabbits using X-ray imaging. The study was carried out by administering the drug-loaded M-GRDDS beads to six healthy New Zealand white rabbits weighing approximately 2–2.5 kg. Prior to the study, the rabbits were kept on a fast overnight and allowed only water. High-density BaSO_4_ (4.4777 g/cm^3^) was used to make the beads radio-opaque. The mucoadhesive beads were prepared by replacing the drug with BaSO_4_ to enhance the X-ray visibility. The X-ray photographs were taken for the rabbits before administering the BaSO_4_-loaded mucoadhesive beads to confirm that no radio-opaque substance was present inside the stomach, and these photographs served as a control. The beads were then administered using a hollow polythene tube with 3–4 mL of water. The X-ray photograph of the rabbits was taken at 0, 1, 3, and 8 h time points. The institutional animal ethics committee of Kasturba Medical College, Manipal, granted approval for the study, IAEC/KMC/68/2023 dated 25.08.2023, MAHE.

### 2.5 *In Vivo* Tissue Distribution Study in the Gastric Mucosa of Rats

For evaluating the enhanced local bioavailability of AMO, MTZ, and PIP, from the prepared mucoadhesive-GRDDS beads, an *in vivo* tissue distribution study in the gastric mucosa was performed on Sprague–Dawley (SD) rats (body weight 200–250 g). The animals were housed in standardized conditions, with a 12 h light/dark cycle (22 ± 2 °C; 50 ± 20% RH). The Institutional Animal Ethics Committee (IAEC) approval number IAEC/KMC/04/2022 approved the study protocol, dated 21.01.2022. To check the local bioavailability of the drugs in the gastric mucosa, a study was performed on two groups of rats (*n* = 3), for the formulation and the free drug. Single dose of the formulation and the free drug were administered after allometric conversion of the human dose: AMO 33.33 mg/kg body weight, MTZ 16.66 mg/kg body weight, PAN 1.3 mg/kg body weight, and PIP 3.33 mg/kg body weight.^34^ Due to its greater metabolic rate and quicker drug clearance than humans, rats require a dosage that is physiologically appropriate, which is ensured by this modification.^4^ Animals from each group were sacrificed by cervical dislocation at the time points 0, 0.25, 0.5, 1, 2, 4, 8, 12, and 24 h, and the gastric tissue was separated out. The gastric mucosa was collected by scraping the surface with a glass slide. The gastric mucosa sample was stored at −80 °C until further analysis. For the sample preparation, 1 g of gastric mucosa was taken and homogenized in 2 mL of water. The IS ceftiofur was added and vortexed for 15 min. After the mixing of the sample, 500 μL of chilled methanol was added for the precipitation. The resultant sample was centrifuged at 10,000 rpm for 15 min at 4 °C. The supernatant was carefully collected and analyzed using the in-house developed LC-MS bioanalytical method.

### 2.6 *In Vivo* Efficacy Evaluation

*H. pylori* were cultured in liquid medium made of Brucella broth and 5% fetal bovine serum (FBS). Antibiotic mix (24 μL) was added to avoid contamination by other microorganisms. To guarantee adequate aeration and nutrient dispersion, the culture was continuously stirred at 1500 rpm for 24 h at 37 °C. Campygen gas-generating sachets (Thermos Scientific, Waltham, Massachusetts, USA) were used to create a microaerophilic atmosphere with a gas composition of 10% CO_2_, 85% N_2_, and 5% O_2_. This was done to replicate the gastric environment’s low-oxygen conditions, which are crucial for the best growth of *H. pylori*.^4^

SD rats were selected for the study based on their well-established use in gastrointestinal research, including *H. pylori* colonization studies. Although mice are more commonly used, SD rats offer advantages such as larger gastric tissue surface area, ease of handling, and better tolerance to repeated sampling procedures. The SD rat model is commonly used because of its physiological relevance and reliability in investigating chronic *H. pylori* infection. Male SD rats weighing 180–200 g were chosen as the study’s experimental participants. A 12 h light/dark cycle, a regulated ambient temperature at 22 ± 2 °C, and a relative humidity of 50 ± 20% were among the normal laboratory conditions in which the animals were kept. Water and food were always accessible throughout the acclimatization process. Water was left accessible to prevent dehydration, but food was removed 24 h before the experimental procedures. Table 1 lists the treatment groups.

**Table 1:** Treatment Groups for the *In Vivo* Evaluation of Efficacy

Group	Treatment	Number of animals
Group 1	Healthy control	6
Group 2	Positive control (Untreated *H. pylori*-infected animals)	6
Group 3	Treatment with free drugs (AMO, MTZ, and PAN)	6
Group 4	Treatment with free drugs + PIP	6
Group 5	Treatment with blank formulation	6
Group 6	Treatment with drug loaded mucoadhesive-GRDDS formulations of AMO, MTZ, and PAN	6
Group 7	Treatment with drug loaded mucoadhesive-GRDDS formulations of AMO, MTZ, PIP, and PAN	6

A chronic infection model was established in SD rats using the *ATCC 700392* strain of *H. pylori*. To develop the infection, 1 mL of bacterial suspension with 5 × 10^–10^ CFU/mL was given orally by gavage twice a day for 3 days in a row. CFU was measured from stomach tissue samples taken 2 and 3 weeks after injection to validate the bacterial model and to confirm infection. Treatment began after a successful infection and lasted for 14 days.^48,49^ The treatment and control groups are provided in Table 1. Dose of the drugs for the efficacy study was selected as per the allometric conversion of the human dose: AMO 33.33 mg/kg body weight, MTZ 16.66 mg/kg body weight, PAN 1.3 mg/kg body weight, and PIP 3.33 mg/kg body weight.^34^ The rats were sacrificed by cervical dislocation after 48 h of the last treatment. The stomachs of animals were removed and cleansed with PBS. The organ was split into two equal longitudinal halves by its primary curvature. One portion of the stomach was used to check the CFU of the infection and another portion of the stomach was used for the histological, biochemical, and gene expression studies.

#### 2.6.1 CFU Determination

One portion of the stomach was homogenized in 1 mL of sterile PBS. Serial dilutions were performed to quantify bacterial load. Tissue homogenate (1 mL) was mixed with 9 mL of fresh Brucella broth in a test tube (1:10 dilution), followed by further dilutions ranging from 10^–2^ to 10^–9^. From each dilution, 1 mL was inoculated onto blood agar plates, which were then incubated under microaerophilic conditions in a trigas incubator (Heal Force Bio-Meditech Holdings Ltd., China) at 37 °C for 3–5 days. Number of colony-forming units (CFU) were determined as per eq 5

5 $$\frac{\mathrm{CFU}}{g}=\frac{\mathrm{Number}\;\mathrm{of}\;\mathrm{Colonies}\times \mathrm{Dilution}\;\mathrm{Factor}}{\mathrm{Volume}\;\mathrm{plated}}\times \frac{1}{\mathrm{Sample}\;\mathrm{Weight}\left(g\right)}$$

#### 2.6.2 Urease Test

Urease reaction test was used to confirm the presence of *H. pylori* infection in the gastric tissue.^3^ A portion of homogenized stomach (100 μL) was added to the urea broth and incubated at 37 °C. Urea broth was prepared as per the manufacturer’s instructions, and the pH of the broth was maintained at 6.7. After the addition of the sample to the urea broth, the sample was incubated and observed for the color change. A shift from yellow to pink color due to ammonia production, typically within 1–3 h is indicative of a positive response to the bacterial load. [Preparation of urea broth: Urea broth contains urea (2%), sodium chloride (0.5%), monopotassium phosphate (0.2%), peptone (0.1%), dextrose (0.1%), and phenol red (0.001%). All components except urea were weighed, added to a conical flask, and dissolved in water. The pH was adjusted, and then the media was autoclaved. A urea solution was prepared accordingly and added to the media after cooling through a sterile filter.

#### 2.6.3 Biochemical Evaluations

The concentration of the inflammatory indicators such as cyclooxygenase-2 (COX-2), tumor necrosis factor-alpha (TNF-α), and Interleukin-1 beta (IL-1 β) in gastric homogenates was determined using commercial ELISA kits developed specially for rats. The 100 mg of stomach tissue was homogenized in 1 mL of RIPA buffer for the extraction of protein from the stomach tissue. The homogenate was centrifuged at 12000*g* for 20 min at 4 °C, and the protein was collected from the supernatant. The supernatants were used for the ELISA analysis. The collected protein was quantified using the BCA protein assay. After the quantification of the protein, the tissue homogenate was aliquoted for a protein concentration of 500 μg/mL. This sample was then analyzed using the respective ELISA kit as per the standard manufacturer’s protocol at a detection wavelength of 450 nm with BioTek synergy H1 microplate reader. The study was performed in triplicate, and the concentration of protein biomarkers was measured in picograms per mL.^4^

#### 2.6.4 Gene Expression Studies

Gene expression analysis provides information on the molecular mechanisms underlying tissue damage, inflammation, and healing. TNF-α and COX-2 are inflammatory proteins released in response to various inflammatory environments. The expression of gene TNF-α and COX-2 was checked in the gastric tissue. An overexpression of TNF-α and COX-2 genes correlates with increased inflammatory response.^50^ cDNA synthesis and quantitative real-time PCR (RT-PCR) were performed to check the gene expression.

For the RNA isolation, 100 mg of the gastric tissue was taken and homogenized in 1 mL tri reagent to extract RNA from the homogenate. To separate the RNA-containing layer from the tissue homogenate, 200 μL of chloroform was added and centrifuged at 12,000 × g for 20 min at 4 °C. The supernatant was collected, added an equal volume of isopropyl alcohol to precipitate the RNA, and centrifuged at 12,000*g* for 20 min at 4 °C. The resultant pellet was washed thrice with 70% ice cold ethanol to eliminate residual salts and contaminants. The RNA pellet was air-dried to eliminate ethanol residues and reconstituted in 20 μL nuclease-free water. The RNA was quantified with a BioTek Synergy H1 microplate reader (Agilent Biotek, Santa Clara, CA, USA).

The isolated RNA from each sample (200 ng) was reverse transcribed into cDNA and quantified by RT-PCR (Thermofisher Quant Studio 5) to target a specific gene by following the kit-based standard procedure (DSS Takara Bio,). The specific primers were created using Primer 3 software (Sigma-Aldrich) for the inflammatory markers TNF-α (Forward: ATGGGCTCCCTCTCATCAGT, Reverse: GGCTGGGTAGAGAACGGATG), COX-2 (Forward: TCTCCTACTACACCAGGGCC, Reverse: ACTCTGTTGTGCTCCCGAAG) and Glyceraldehyde 3-phosphate dehydrogenase (GAPDH) (Forward: CTCGTGGTTCACACCCATCA, Reverse: CTCGTGGTTCACACCCATCA). GAPDH worked as a standard to normalize the gene expression levels.^50^

#### 2.6.5 Histopathological Evaluation

The histopathology study was conducted on the dissected portion of the stomach on the day of the animal’s sacrifice and fixed in 10% formalin solution for 24 h. The tissue was then dehydrated in ascending grades of alcohol and embedded in paraffin. 24 h after block preparation, paraffin sections were obtained on clean glass slides and stained with hematoxylin and eosin stains. The slides were observed for histopathological changes under a light microscope. In the microscope, the ulcer area reduction, mucosal integrity, and signs of fibrosis and necrosis were evaluated. Relevant photomicrographs were taken.^51^

### 2.7 Stability Study of Beads at Intermediate and Accelerated Conditions

Stability of the drug loaded mucoadhesive-GRDDS beads was determined at two ICH conditions.^52^ Intermediate (30 ± 2 °C/65 ± 5% RH) and accelerated conditions (40 ± 2 °C/75 ± 5% RH). The sampling time points were 0, 1, 3, and 6 months. The % degradation of drugs was calculated.

## 3 Results and Discussion

### 3.1 Characterization Data of Drug-Loaded Mucoadhesive GRDDS Beads of Amoxicillin, Metronidazole, Piperine, and Pantoprazole

#### 3.1.1 Appearance and Size of the Prepared Beads

The mucoadhesive GRDDS beads loaded with AMO, MTZ, PAN, and PIP were spherical, light brownish, elastic, and of soft texture. A photograph of the prepared beads is presented in Supplementary Figure 1. The average size of the dried beads determined using the stage micrometer ranged from 1.13 ± 0.05 mm to 1.39 ± 0.07 mm (AMO beads: 1.39 ± 0.07 mm; MTZ beads: 1.27 ± 0.11 mm; Pan beads: 1.32 ± 0.09 mm; PIP beads: 1.13 ± 0.05 mm).

#### 3.1.2 Drug Entrapment Efficiency of Drug-Loaded M-GRDDS Beads

All the drug loaded M-GRDDS beads showed promising entrapment efficiency of above 80% (AMO beads: 85.26 ± 3%; MTZ beads: 81.53 ± 3%; PAN beads: 80.65 ± 4%; PIP beads: 88.23 ± 3%). Different polymer ratios were used to achieve the highest entrapment efficiency while maintaining the integrity of the beads (details of trials are provided in Supplementary Tables S1–S4). The findings of the trials showed that, with an increase in the amount of polymer, the entrapment efficiency of the drug also increased. This increased entrapment efficiency supports sustained drug release, improved mucoadhesion, and higher drug concentrations at the target site, all of which are crucial for effectively eradicating *H. pylori*. Similar results were reported by Sen et al., 2023 from the alginate-based gastroretentive drug delivery systems demonstrating entrapment efficiencies exceeding 85%.^53^

#### 3.1.3 % Swelling of Drug-Loaded M-GRDDS Beads

Swelling behavior of the drug loaded M-GRDDS beads is illustrated in Figure 2. AMO-loaded beads exhibited 207% and 175% swelling at pH 1.2 and 7.4, respectively, after 6 h. MTZ-loaded beads showed 192% and 169% swelling at pH 1.2 and 7.4, respectively. Swelling of PIP-loaded beads was 196% and 150% at pH 1.2 and 7.4, respectively. PAN-loaded beads achieved 180% swelling at pH 7.4. The swelling behavior of the beads, characterized by the higher swelling at acidic pH (1.2) compared to the near-neutral pH (7.4), can be attributed to the hydration and protonation of the polymeric network, forming a gel barrier that regulates drug diffusion. Chitosan, being a cationic polymer, swells more under acidic conditions due to protonation of amino groups, inducing electrostatic repulsion, while the subsequent decrease in swelling after 6 h is likely due to polymer erosion. This pH-responsive swelling and erosion profile aligns well with the mechanisms described by Rizwan et al., 2017, who detailed similar behavior in pH-sensitive hydrogels, and Tripathi et al., 2019, who emphasized the role of gastroretentive systems in enhancing localized drug release and mucoadhesion for effective *H. pylori* treatment.^54,55^

**Figure 2:**
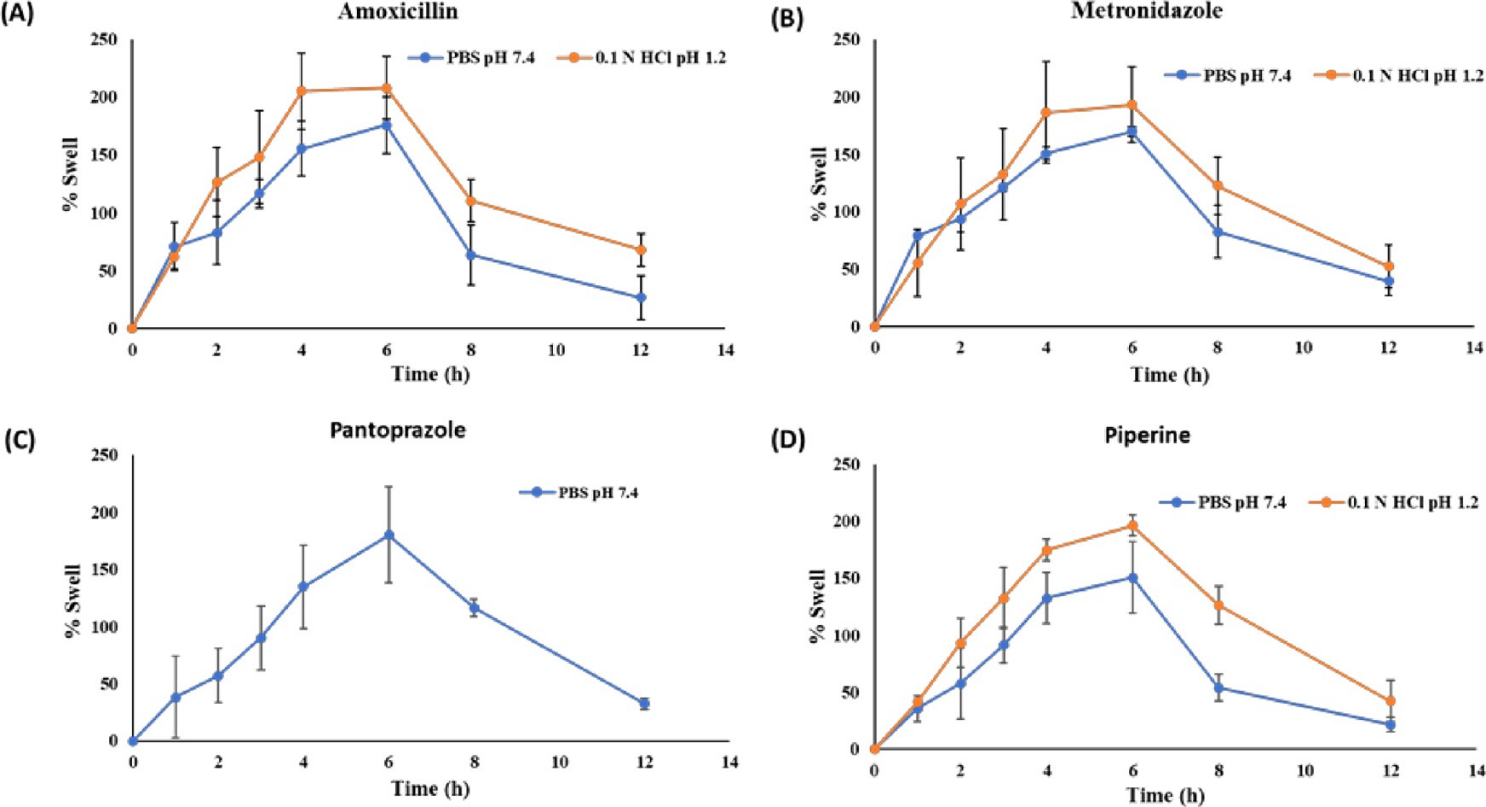
Swelling behavior of the prepared drug-loaded M-GRDDS beads (A) amoxicillin, (B) metronidazole, (C) pantoprazole, and (D) piperine.

#### 3.1.4 Surface Morphology of the Drug-Loaded M-GRDDS Beads

Scanning Electron Microscope (SEM) analysis was used to determine the beads’ shape, texture, porosity, and roughness of the drug loaded M-GRDDS beads. These parameters greatly impact their mucoadhesive qualities and stomach retention. A rough or porous surface improves mucoadhesion by providing a larger surface area for interaction with the mucosal lining. Furthermore, SEM helps to analyze the uniformity of the beads’ size and shape, which is crucial for accurate drug release. SEM can verify the consistency and integrity of the coating layer and helps in establishing an association between the functional behavior and physical attributes of the GRDDS beads, ensuring formulation stability and efficacy. For the GRDDS beads, the particle size evaluation with SEM is crucial since it directly impacts performance characteristics like drug release, mucoadhesion, gastric retention, and floating behavior. SEM offers a clear visualization of individual beads, enabling in-depth evaluation of particles’ size, shape, and surface structure at the microscale level. The homogeneously shaped beads adhere to the gastric mucosa and show longer gastric residence. Additionally, consistent particle size guarantees better batch-to-batch reproducibility, consistent drug distribution, and predictable *in vivo* behavior.

The SEM analysis showed that the prepared beads are spherical in shape. This spherical shape is a result of ionic gelation between sodium alginate and calcium chloride. When negatively charged sodium alginate chains come in contact with positively charged calcium ions, it instantly cross-link by rapid electrostatic interaction, forming a stable, water-insoluble gel network. George and Abraham, and Hariyadi and Islam, also support that cross-linking density and bead morphology in alginate-based systems strongly depend on ionic interactions and polymer concentration.^56,57^ SEM analysis further confirms that the concentration of sodium alginate plays a crucial role in the structure of the bead network. The beads’ SEM photograph (Figure 3) showed that the particle size of AMO, MTZ, PAN, and PIP beads was 1.16 mm, 1.24 mm, 1.13 mm, and 1.35 mm, respectively. Abourehab et al., also reported similar spherical morphologies with controlled particle dimensions, highlighting ionic cross-linking conditions in achieving a reproducible bead size.^58^ The SEM analysis in this study revealed a porous and rough surface topology, providing enhanced surface area for interaction with the gastric mucosa and thereby promoting mucoadhesion. Lopes et al., also demonstrated similar findings, showing that rough bead surfaces improve mucoadhesive properties.^59^ Das and Senapati, observed that increased porosity not only aids adhesion but also facilitates earlier hydration and swelling, leading to modulated drug release kinetics, a phenomenon evident in our study as well.^60^ SEM observations support the findings of Bennacef et al., which support structural stability, mucoadhesive potential, and sustained release capability of the developed M-GRDDS beads.^61^

**Figure 3:**
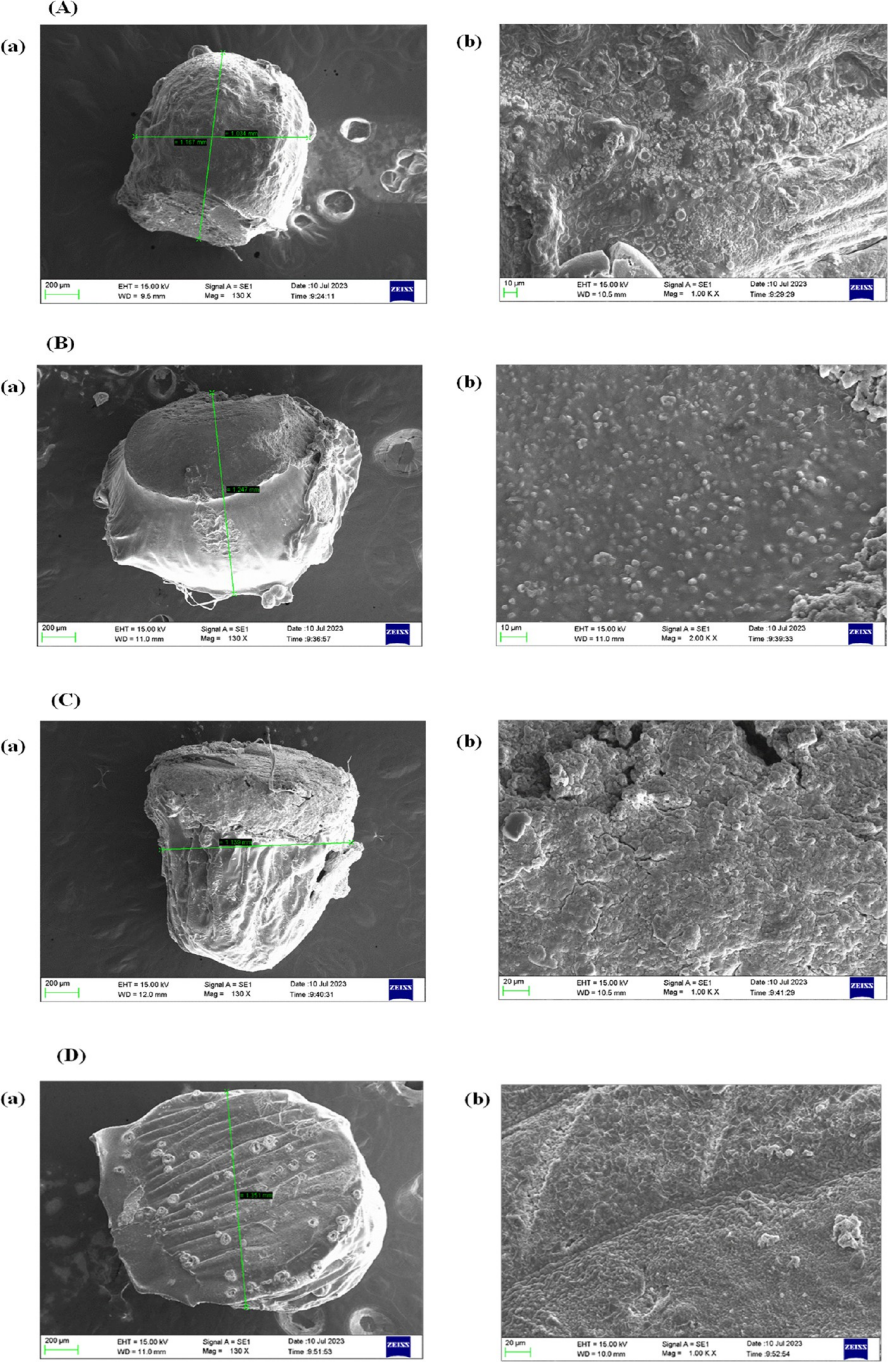
SEM images of the prepared drug-loaded M-GRDDS beads (A) amoxicillin, (B) metronidazole, (C) pantoprazole, and (D) piperine; showing two key structural views: (a) size and morphology: zoomed-in view of individual beads, clearly showing their overall dimensions, (b) surface texture: depicting the highly porous surface structure of the beads.

#### 3.1.5 Fourier Transform Infrared Spectroscopy (FTIR) Analysis

The chemical interaction between the selected drugs and the polymers used in the beads was evaluated using FTIR spectroscopy. The FTIR spectra of pure drugs, placebo, and drug-loaded beads were compared. The respective spectra obtained is provided in Figure 4. The characteristic bands of pure AMO were seen at 3158.68 cm^–1^ for phenol OH stretching, 3027.75 cm^–1^ for benzene ring CH stretching, 2966.82 cm^–1^ for methyl CH stretching, 2357.01 cm^–1^ for COO^–^ symmetric stretching, 1770.45 cm^–1^ for β-Lactam CO stretching, 1683.65 cm^–1^ for amide I and CO stretching, 1575.34 cm^–1^ for benzene ring C=C stretching, 1316.59 cm^–1^ for fused thiazolidine β-Lactam ring, and 1245.83 cm^–1^ for amide III, NH band CN stretching. In the case of pure MTZ, the characteristic bands were seen at 1070.71 cm^–1^ for C–N stretching, 1531.93 cm^–1^ for N=O stretching, 3095.28 cm^–1^ for C–H stretching, 1475.15 cm^–1^ for C=C stretching, 3206.88 cm^–1^ for O–H stretching, 1579.95 cm^–1^ for N–N bending, and 1264.01 cm^–1^ for C–O stretching. For PAN, the bands were at 3552.47 cm^–1^ for N–H stretching, 1587.12 cm^–1^ for C–O stretching, 1360.13 cm^–1^ for C–F, 1164.30 cm^–1^ for C–O (aromatic) stretching, and 1035.40 cm^–1^ for C–S stretching. For PIP they were at 578.56 cm^–1^ for symmetric and asymmetric stretching of C=C (diene), 1487.40 cm^–1^ for aromatic stretching of C=C (benzene ring), 1631.28 cm^–1^ for stretching of −CO–N, 1436.56 cm^–1^ for CH_2_ bending, 922.13 cm^–1^ for C–O stretching, 1250–1190 cm^–1^ for asymmetrical stretching = C–O–C, and 840.96 cm^–1^ for C–H bending. These chrematistic bands of pure drugs showed the purity of the drugs. The bands obtained for the prepared placebo beads were at 3354.22 cm^–1^ for O–H stretching, 1613.87 cm^–1^ for C–O stretching, 1426.53 cm^–1^ for COO-(symmetric), and 1080.31 cm^–1^ for C–O–C groups from the polymer. After the loading AMO in the beads, the bands were seen at 3353.30 cm^–1^ for O–H stretching, 1613.84 cm^–1^ for C–O stretching, 1426.11 cm^–1^ for COO–(symmetric), and 1080.56 cm^–1^ for C–O–C groups. In case of MTZ-loaded beads, the bands were at 3552.12 cm^–1^ for N–H stretching, 1614.56 cm^–1^ for C–O stretching, 1424.31 cm^–1^ for C–F, 1080.82 cm^–1^ for C–O (aromatic) stretching, and 1024.47 cm^–1^ for C–S stretching. For the PAN-loaded beads, they were at 3552.03 cm^–1^ for N–H stretching, 1613.56 cm^–1^ for C–O stretching, 1426.81 cm^–1^ for C–F, 1080.07 cm^–1^ for C–O (aromatic) stretching, and 1023.91 cm^–1^ for C–S stretching. The characteristic bands of PIP-loaded beads were at 3552.88 cm^–1^ for N–H stretching, 1613.38 cm^–1^ for C–O stretching, 1426.36 cm^–1^ for C–F, 1080.41 cm^–1^ for C–O (aromatic) stretching, and 1024.06 cm^–1^ for C–S stretching. The FTIR spectra of drug-loaded beads typically show the disappearance or significant reduction of characteristic peaks of pure drugs, indicating strong intermolecular interactions such as hydrogen bonding or ionic bonds between the drugs and the polymer matrix (e.g., sodium alginate and chitosan). This suggests homogeneous dispersion and effective entrapment of drugs within the polymer network, which prevents crystallization and enhances stability. Such molecular interactions are crucial for promoting sustained drug release and improving mucoadhesive properties. These findings align with the observations of Sougandhi et al., who demonstrated that similar interactions play a key role in governing stability and functional performance.^62^

**Figure 4:**
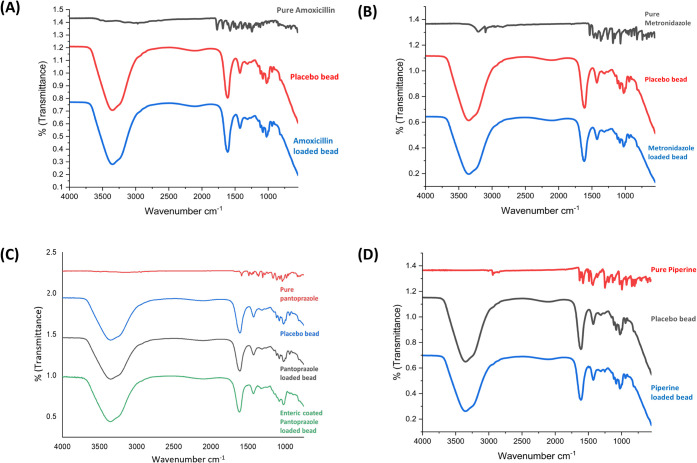
FTIR spectra of pure drug, placebo beads, and drug-loaded M-GRDDS beads (A) amoxicillin, (B) metronidazole, (C) pantoprazole, and (D) piperine.

#### 3.1.6 X-ray Diffraction (XRD) Analysis

XRD analysis revealed the peaks corresponding to the crystalline nature of AMO, MTZ, PAN, and PIP drugs. Each drug showed (Figure 5) specific intense peaks at various 2θ angles, indicating their unique crystalline structure: AMO showed intense peaks at 2θ of 14.7°, 17.6°, 18.9°, 22.7°, 26.3°, 28.3°, and 31.5°; MTZ at 2θ of 11.9°, 24.4°, 25.1°, 27.1°, 27.7°, 28.9°, and 29.1°; PAN at 2θ of 16.3°, 20.1°, 21.8°, 22.4°, 24.1°, and 25.9°, and PIP at 2θ of 14.1°, 15.6°, 19.2°, 21.1°, 22°, 25.4°, and 27.8°. XRD diffractograms of pure AMO, MTZ, PAN, and PIP displayed distinct, sharp peaks at their characteristic 2θ angles, confirming their crystalline nature. In contrast, the drug-loaded M-GRDDS beads showed the complete disappearance of these sharp peaks and instead exhibited broad, diffuse patterns similar to placebo formulation, indicating a transformation of the drugs from crystalline to amorphous states within the polymeric network. This amorphization suggests molecular dispersion of the drugs, which is known to enhance solubility and bioavailability by disrupting the crystal lattice and increasing free energy, thereby improving dissolution rates critical for effective gastric drug delivery. Similar observation was reported by Rodríguez et al., showing the absence of crystalline peaks in the polymer-based formulation signifying the successful amorphization of the drug. Stabilizing the drugs in an amorphous form within the formulation supports sustained and controlled release, an essential feature for localized gastric therapies such as *H. pylori* eradication.^63^

**Figure 5:**
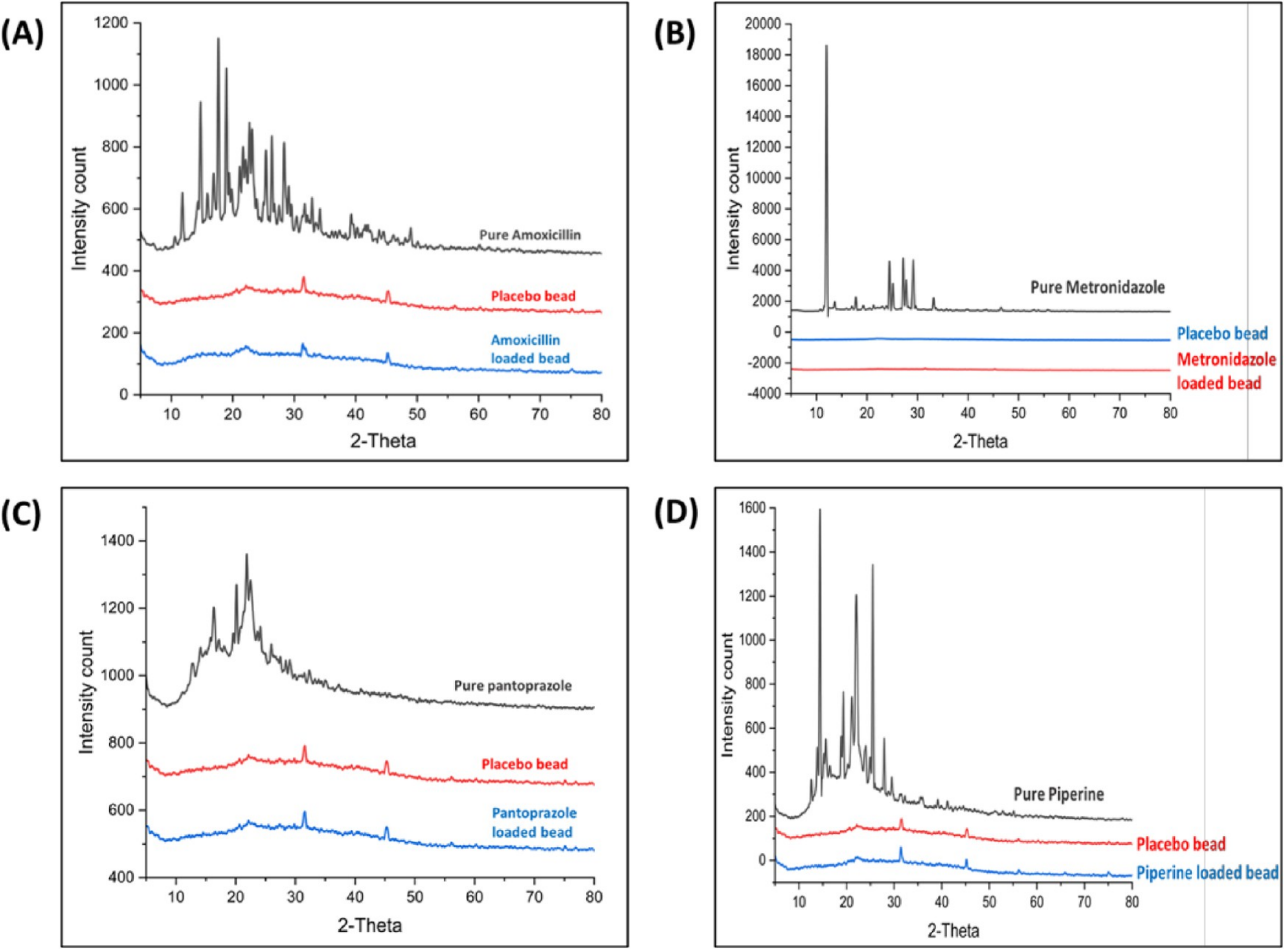
XRD diffractogram of pure drug, placebo, and drug-loaded M-GRDDS beads of (A) amoxicillin, (B) metronidazole, (C) pantoprazole, and (D) piperine.

#### 3.1.7 Differential Scanning Calorimetry (DSC) Analysis

The thermodynamic properties and interactions of AMO, MTZ, PAN, and PIP when encapsulated in M-GRDDS beads were analyzed from the DSC thermograms (Figure 6). A successful encapsulation is evidenced by the absence of prominent drug peaks in the thermograms of the drug-loaded beads, which show the drugs are fully incorporated within the polymer matrix. The free drug of AMO exhibited an endothermic peak on the DSC thermogram at 132.73 °C. On the other hand, the AMO-loaded M-GRDDS beads displayed a slightly lower peak at 125.43 °C, similar to the placebo beads, which showed a peak at 125.78 °C. The pure form of MTZ showed an endothermic peak at 170.34 °C, while the MTZ-loaded beads showed the peak at 128.83 °C, similar to the peak seen for placebo beads at 125.78 °C. PAN and PIP also showed a similar pattern of changes in the thermogram when loaded into the polymeric matrix. The DSC thermograms of pure AMO, MTZ, PAN, and PIP exhibited distinct melting endothermic peaks, confirming their crystalline nature. In contrast, these characteristic transitions were either significantly reduced or completely absent in the drug-loaded alginate beads. The thermal profiles of the loaded formulations closely resembled those of placebo beads, indicating successful drug encapsulation and conversion of the crystalline drugs into amorphous states within the polymeric matrix. Such amorphization is advantageous as it enhances solubility and dissolution rates, ultimately contributing to improved bioavailability and more efficient drug delivery. These thermal changes also suggest strong drug–polymer interactions that limit crystallinity, thereby supporting sustained release and gastric-targeted delivery. These findings are consistent with earlier reports on polymer-based formulations by Saha and Ray.^64–66^

**Figure 6:**
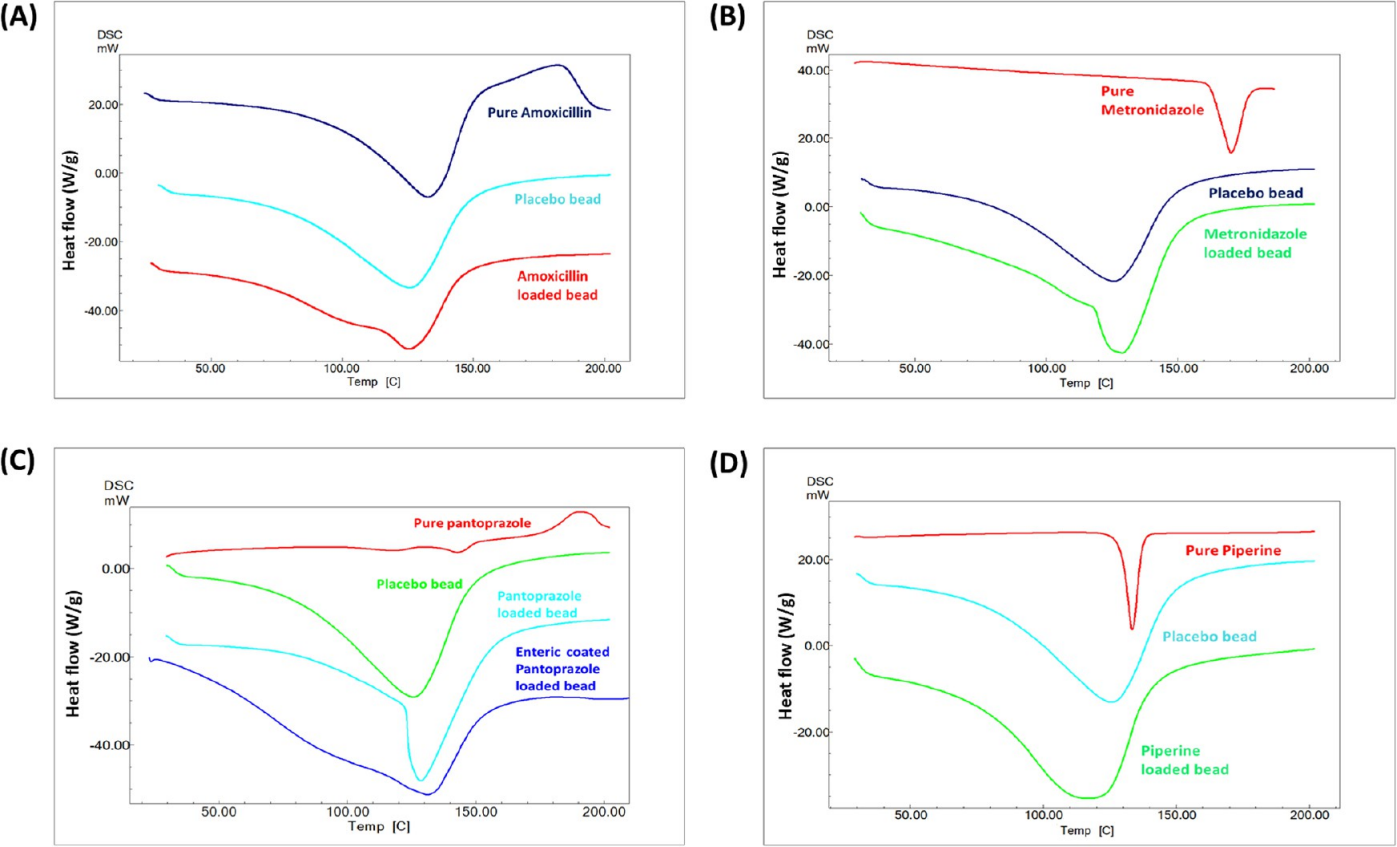
DSC thermogram of pure drug, placebo beads and drug loaded M-GRDDS beads of (A) amoxicillin, (B) metronidazole, (C) pantoprazole, and (D) piperine.

#### 3.1.8 *In Vitro* Floating Study

A floating study was performed to ensure that the prepared beads do not float in the stomach under certain conditions. The *in vitro* floating behavior of mucoadhesive beads was evaluated through visual observation under controlled circumstances. The observations suggested that all the beads sank to the bottom of the beaker within 3 min of introduction in both the acidic and alkaline pH conditions. No floating was observed. This immediate sinking indicates that the formulation does not remain buoyant. Such behavior is in agreement with earlier findings by Adebisi et al., who reported that nonfloating mucoadhesive beads achieved prolonged gastric retention through intimate mucus adherence instead of flotation.^65^ Similarly, Shu et al., emphasized that biomaterial-based gastric delivery systems can effectively exploit mucoadhesion to enhance localized drug residence. The reduced floating time displayed by our beads is particularly advantageous for *H. pylori* eradication, as it prolongs contact time with the stomach lining and facilitates localized, sustained drug delivery at the infection site.^66^

#### 3.1.9 *In Vitro* Mucoadhesive Potential

The mucoadhesive property of the prepared beads was evaluated by the *in vitro* wash-off method. Results are presented in Table 2. The observation showed that the mucoadhesive GRDDS beads showed better mucoadhesion at pH 1.2 than at pH 7.4. Mucoadhesion of the drug loaded M-GRDDS beads is significantly stronger in acidic conditions of the stomach because of the pH-dependent ionization and electrostatic interactions of the polymer with the gastric mucin. In acidic conditions (pH 1.2), chitosan, a cationic polymer (p*K*_a_ ∼ 6.5), maintains its protonated (+ve charge) state, which exhibits electrostatic attraction to the negatively charged mucin in the stomach mucosa and enhances adhesion. On the other hand, chitosan breaks down at pH 7.4 (intestinal environment), which lowers its positive charge and lessens its ability to interact with mucin. By creating a gel layer at low pH, sodium alginate, an anionic polymer, also contributes to adhesion and strengthens mucoadhesion. On the other hand, alginate stays ionized and expands excessively at pH 7.4, resulting in decreased adhesion and loss of compactness. Furthermore, mucin itself varies structurally at different pH values; at neutral pH it becomes less interacting and more hydrated, which weakens mucoadhesion even further. Thus, compared to neutral pH (7.4), the mucoadhesion of chitosan-alginate beads showed better mucoadhesion at acidic pH (1.2). The *in vitro* mucoadhesion results confirmed that the cationic nature of chitosan is strengthened in acidic conditions, enabling strong electrostatic interaction with negatively charged gastric mucin and enhancing adhesion similar to that reported by Lopes et al., 2016.^59^ At neutral pH (7.4), chitosan loses its positive charge while alginate swells, leading to weaker mucoadhesion. This pH-dependent adhesion behavior aligns with earlier evidence on chitosan–alginate systems fabricated by Li et al.^67^ Such properties make these beads highly suitable for gastric-specific drug delivery, ensuring prolonged retention and targeted action against *H. pylori*.

**Table 2:** *In Vitro* % Mucoadhesion of the Drug-Loaded M-GRDDS Beads

	% Mucoadhesion	
Drug	pH-1.2	pH-7.4
Amoxicillin	85%	72%
Metronidazole	80%	66%
Pantoprazole	23%	78%
Piperine	87%	75%

#### 3.1.10 *In Vitro* Drug Release

The release pattern of drugs from the prepared mucoadhesive beads exhibited a biphasic behavior, where an initial burst release was observed, followed by a gradual and sustained drug release phase, extending up to 8 h as showed in the Figure 7. At pH 1.2, the beads released 89.69% of AMO and 91.41% of MTZ after 8 h, showing a higher solubility and release efficiency in an acidic environment. However, at pH 7.4, the drug release was comparatively lower, with 57.83% of AMO and 75.77% of MTZ released over the same period. Similarly, PIP release was 89.12% at pH 1.2 and 81.71% at pH 7.4, indicating its moderate solubility in both acidic and neutral environments. In contrast, PAN showed 89.33% release at pH 7.4, highlighting its stability and optimized releases in the simulated intestinal medium. The results demonstrated that the alginate beads coated with chitosan successfully delivered sustained drug release, providing extended therapeutic efficacy which will reduce premature drug degradation and improve stomach retention. This mucoadhesive-GRDDS system has the potential to optimize localized drug administration, improve bioavailability, and improve patient compliance, as indicated by the observed pH-dependent drug release behavior. The *in vitro* release studies of AMO, MTZ, PAN, and PIP-loaded beads exhibited a biphasic release profile characterized by an initial burst followed by a sustained diffusion-controlled release from the chitosan–alginate matrix extending up to 8 h. Similar biphasic behavior was reported by Li et al., where chitosan–alginate nanoparticles showed rapid initial release of surface-bound drug followed by controlled diffusion through the polymeric matrix.^67^ In our study, the higher release of AMO, MTZ, and PIP in acidic medium (pH 1.2) is attributed to their greater solubility and the protonation of chitosan, which enhances polymer hydration and drug diffusion under gastric conditions, thereby supporting localized delivery to the stomach mucosa against *H. pylori*. In contrast, PAN displayed optimized release at pH 7.4 due to its acid-labile nature, supporting its protection in gastric fluid and targeted intestinal delivery. This pH-dependent behavior confirms the suitability of the developed GRDDS beads for site-specific release, improved stability, reduced drug degradation, and prolonged gastric residence.

**Figure 7:**
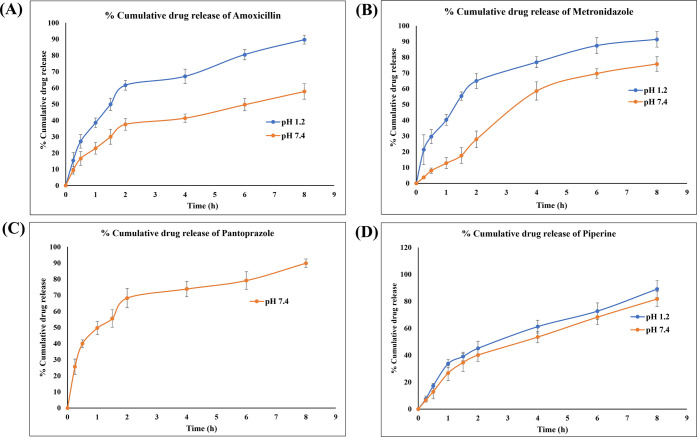
*In vitro* drug release profile of drug loaded M-GRDDS beads at pH 1.2 and 7.4 (A) amoxicillin, (B) metronidazole, (C) pantoprazole, and (D) piperine.

#### 3.1.11 *In Vitro* Release Kinetics

A curve-fitting approach was used to investigate the drug release kinetics using mathematical models, such as zero order, first order, Higuchi, Korsmeyer-Peppas, and Hixson-Crowell models. The findings, which are compiled in Table 3, provide insight into the primary release mechanism that controls each drug at various pH values (1.2 and 7.4). The release kinetics investigation results showed that each drug had a specific kinetic profile, as reveal by the correlation coefficient (*R* ^2^) values. At pH 1.2 and 7.4, AMO displayed Higuchi and Korsmeyer-Peppas model, demonstrating a non-Fickian drug release affected by diffusion processes. MTZ-loaded beads followed the Higuchi model at pH 7.4 and Korsmeyer-Peppas model at pH 1.2. PAN-loaded beads, which followed the Higuchi model at pH 7.4, showed sustained release in the alkaline medium. PIP demonstrated Higuchi and Korsmeyer-Peppas release profile at pH 1.2 and pH 7.4, indicating a diffusion-controlled release mechanism consistent with PIP’s hydrophobic characteristics and steady diffusion through the polymer matrix. These results demonstrate the importance of formulation design in maximizing drug release behavior according to pH conditions and drug characteristics, ensuring higher bioavailability and therapeutic effectiveness. Optimizing sustained drug delivery and limiting dosage variations are all made easier with an understanding release kinetics. The biphasic behavior of the prepared beads reflects an initial rapid release of surface-bound drug, followed by sustained release mediated by matrix relaxation, swelling, and polymer–drug interactions. Similar findings were reported by Li et al., and Patil et al., where drug release from chitosan–alginate systems followed biphasic kinetics governed by diffusion through the polymer network and polymer swelling.^67,68^ Such model-based analysis confirms the mechanistic basis of drug release and enables precise optimization of formulation design. The *n* value of the release kinetics showed in Table 3 indicates the release mechanism of drugs from the formulation. Values near 0.5 suggest diffusion-controlled release, while higher values imply polymer relaxation. Metronidazole at pH 7.4 shows the highest “*n*” (0.8991), indicating anomalous transport behavior. These results align with findings reported by Thai et al., who emphasized that tailored release kinetics in chitosan–alginate nanoparticles can enhance bioavailability, sustain therapeutic levels, and improve site-specific gastric retention, ultimately supporting more effective management of conditions such as *H. pylori* infection.^69^

**Table 3:** Correlation Coefficient (*R* ^2^) Values and “*n*” Values of Different Kinetic Models of Drug Release

		Correlation coefficient (R^2^)					
					Korsmeyer peppas plot		
Drug name	pH	Zero order plot	First order plot	Higuchi plot	*R* ^2^	*n*	Hixson-Crowell plot
Amoxicillin	pH 1.2	0.815	0.866	0.963	0.955	0.483	0.935
	pH 7.4	0.844	0.813	0.957	0.972	0.495	0.928
Metronidazole	pH 1.2	0.791	0.892	0.752	0.911	0.433	0.727
	pH 7.4	0.850	0.783	0.966	0.472	0.899	0.975
Pantoprazole	pH 7.4	0.711	0.822	0.912	0.260	0.330	0.869
Piperine	pH 1.2	0.906	0.768	0.987	0.945	0.659	0.972
	pH 7.4	0.928	0.685	0.989	0.381	0.695	0.979

### 3.2 *In Vivo* Mucoadhesion Study in Rabbits

Based on the positive results obtained from the *in vitro* mucoadhesion and the release studies of M-GRDDS, an *in vivo* X-ray imaging study was conducted in rabbits to confirm the mucoadhesion. The gastric retention behavior of the drug loaded M-GRDDS beads was examined in the rabbit model, to ensure that they stay in the stomach as expected. X-ray photographs of the rabbit’s stomach taken at 0, 1, 3, and 8 h are shown in Figure 8. As per the study findings, the beads’ mean gastric residence time (GRT) was more than 8 h, suggesting both successful mucoadhesion and prolonged gastric retention. The drug can be continually delivered over a longer period of time because of the prolonged residence of the beads, which guarantees that they can withstand the four phases of stomach emptying. The study revealed the mucoadhesion property of the GRDDS system and proved the beads maintain the structural integrity in the acidic environment. This can help in site-specific absorption in the stomach and regulated release over a lengthy period of time, resulting in improved patient compliance and therapeutic efficacy. Similar results were observed by Sen et al., and Seth et al., where chitosan–alginate–based systems exhibited extended gastric residence and controlled drug release for up to 8–12 h in animal models, attributed to the synergistic effects of mucoadhesion and polymer matrix integrity.^53,70^ In our study, the prolonged gastric retention highlights the ability of these beads to maintain intimate contact with the gastric mucosa, enabling sustained drug release at the absorption site. Such extended retention ensures higher local drug concentrations, improved therapeutic efficacy, and enhanced patient compliance, making mucoadhesive GRDDS systems especially valuable for gastric-specific therapies such as *H. pylori* eradication.

**Figure 8:**
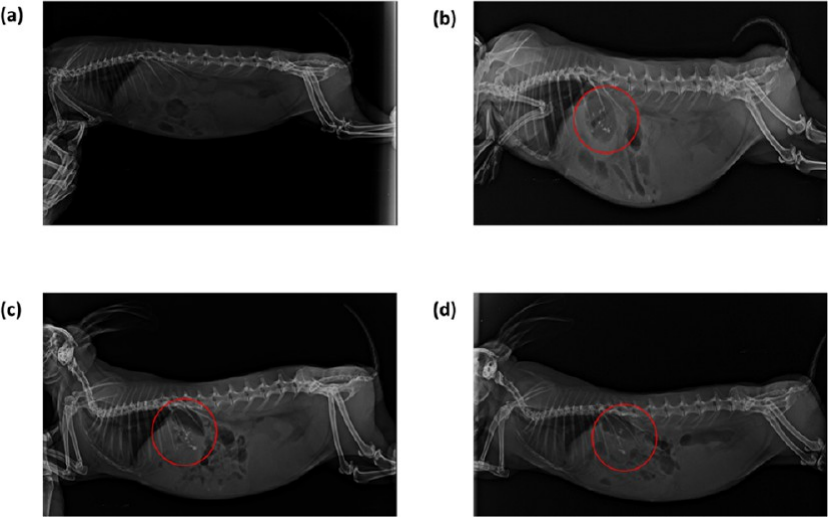
X-ray photographs of a rabbit: (a) Control group, (b) with drug-loaded M-GRDDS beads 1 h, (c) with drug-loaded M-GRDDS beads 3h, and (d) with drug-loaded M-GRDDS beads 8 h.

### 3.3 *In Vivo* Bioavailability in Gastric Mucosal Tissue of Rats

This study was performed to understand the usefulness of the mucoadhesive-GRDDS beads for the local delivery of a drug in the gastric mucosa, where the bacteria reside. Results of the different PK parameters evaluated at the gastric mucosa are shown in Table 4 and Figure 9. Results showed that the drug-loaded M-GRDDS beads released the drugs in the gastric mucosa and their amounts were higher than those available from the administration of free drugs of AMO, MTZ, and PIP. As can be visualized from Figure 9, the AUC_0‑t_ values of drug-loaded beads were higher than those of the free drug. This can be attributed to the higher bioavailability of drugs to the gastric mucosa from the GRDDS system. Other PK parameters such as *C*_max_ and *t*_1/2_ also support this observation. As the formulation adhered to the gastric mucosa, the Tmax was higher for the GRDDS beads than for the free drugs. Increased *C*_max_ and *t*_1/2_ signify the improved availability of drugs in the gastric mucosa. Moreover, the elimination constant of the drug loaded GRDDS formulation was lower than that of the free drugs. Overall, the pharmacokinetic evaluation revealed that mucoadhesive-GRDDS beads significantly enhanced drug retention and bioavailability within the gastric mucosa compared to free drugs, as evidenced by higher AUC_o‑t_, elevated *C*_max_, and prolonged *t*_1/2_ values for AMO, MTZ, and PIP. The extended *T*_max_ observed for the bead formulations further demonstrated the controlled, sustained release behavior and strong mucoadhesive interactions with the gastric mucosal lining. Similar trends were reported by Sahin et al., Jelvehgari et al., and Paul et al., where mucoadhesive systems markedly improved gastric residence time and increased localized drug concentrations at the mucosal site.^71–73^

**Figure 9:**
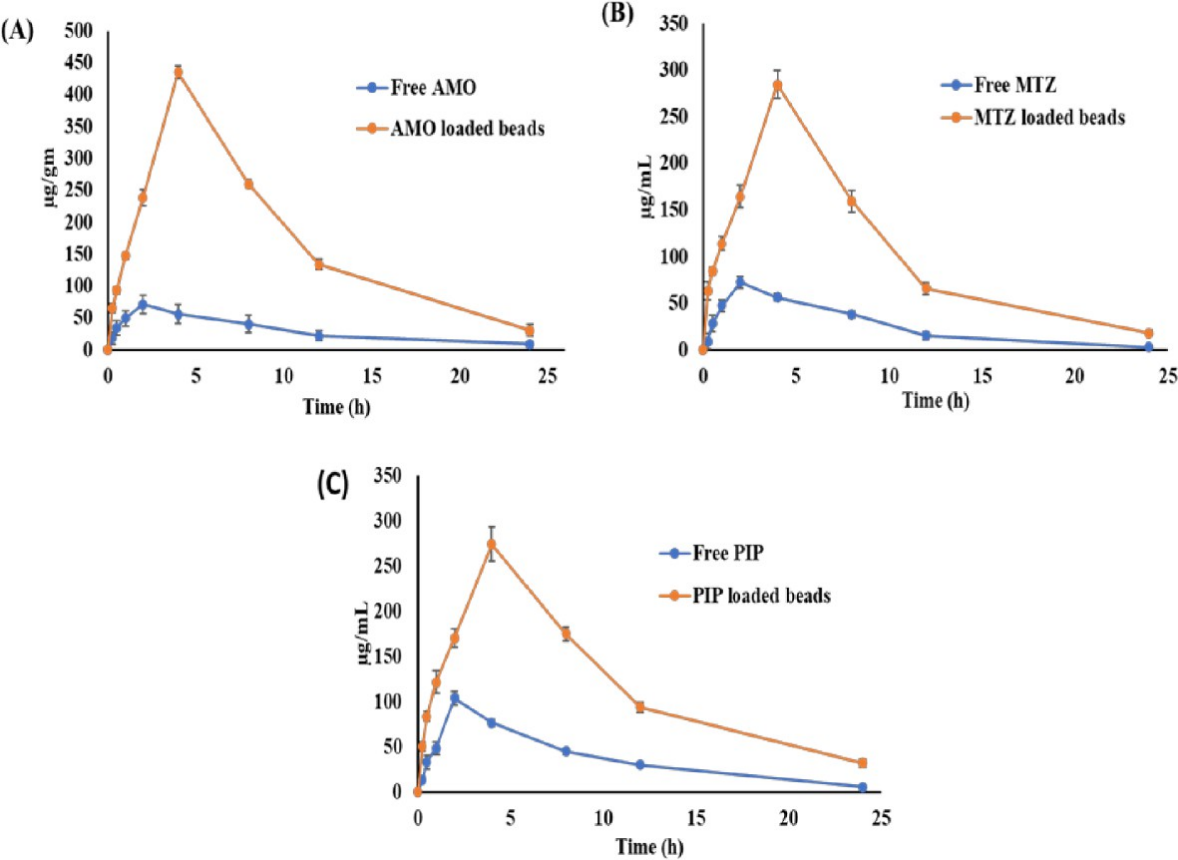
Gastric mucosal bioavailability of drugs from the drug loaded M-GRDDS beads in comparison with free drugs (A) amoxicillin, (B) metronidazole, and (C) piperine.

**Table 4:** PK Profile of Free Drug and Drug-Loaded M-GRDDS Beads in the Gastric Mucosa

PK profile of free drug and drug-loaded M-GRDDS beads in the gastric mucosa						
PK parameters	Free AMO	AMO loaded beads	Free MTZ	MTZ loaded beads	Free PIP	PIP loaded beads
**AUC**_**0**‑**t**_ ***(μg/g × h)***	718.26 ± 219.45	4116.51 ± 146.03	622.47 ± 76.65	2502.11 ± 80.05	894.78 ± 65.71	2804.60 ± 51.80
***C***_**max**_ **(μg/g)**	71.08 ± 14.15	435.37 ± 9.75	72.80 ± 6.53	284.63 ± 15.36	104.46 ± 7.54	274.26 ± 19.01
***T***_**max**_ **(h)**	2 ± 0	4 ± 0	2 ± 0	4 ± 0	2 ± 0	4 ± 0
**Half-life (h)**	11.52 ± 1.15	13.53 ± 2.81	8.10 ± 1.81	9.88 ± 0.78	9.61 ± 1.53	12.98 ± 2.41
**MRT**_**0**‑**t**_ **(h)**	7.76 ± 0.49	7.68 ± 0.25	6.57 ± 0.30	7.22 ± 0.14	7.20 ± 0.29	7.81 ± 0.33
**Ke (h** ^**–** **1**^ **)**	0.06 ± 0.009	0.052 ± 0.01	0.08 ± 0.01	0.07 ± 0.005	0.07 ± 0.01	0.05 ± 0.013

### 3.4 *In Vivo* Efficacy of Drug-Loaded M-GRDDS Beads in Eradication of *H. pylori* Infection

#### 3.4.1 Colony-Forming Units (CFU)

The control group, treated solely with PBS and not infected with *H. pylori*, showed no colony growth, confirming the absence of contamination. The positive control group, infected but untreated, exhibited a bacterial load of 5.4 CFU/g. Group 3, treated with free drugs (AMO, MTZ, and PAN), reduced the bacterial count to 3.4 CFU/g. Adding piperine (PIP) to the free drugs in Group 4 lowered the count to 3.1 CFU/g, suggesting PIP’s role in enhancing drug efficacy, possibly by improving bioavailability or inhibiting bacterial efflux pumps. Group 5, which was treated with placebo beads without drugs, showed a bacterial count of 5.1 CFU/g, indicating a minor nonspecific effect, likely due to the bead material or altered gastric environment. In Group 6, which was treated with the mucoadhesive-GRDDS beads loaded with AMO, MTZ, and enteric-coated PAN, the bacterial load declined significantly to 1.2 CFU/g. The group 7, which was treated with mucoadhesive GRDDS beads loaded with AMO, MTZ, PIP, and enteric-coated PAN was found to be the most successful treatment option with the lowest bacterial count of 0.5 CFU/g. This significant decrease (*p* < 0.05 in comparison to the positive control) indicates the higher efficiency of the formulation in eradicating *H. pylori* infection. Figure 10A shows photographs of the cultured plates of *H. pylori* from the stomach homogenate of the treatment groups. Figure 10B presents the number of colony forming units per gram of the stomach homogenate taken after the treatments.

**Figure 10:**
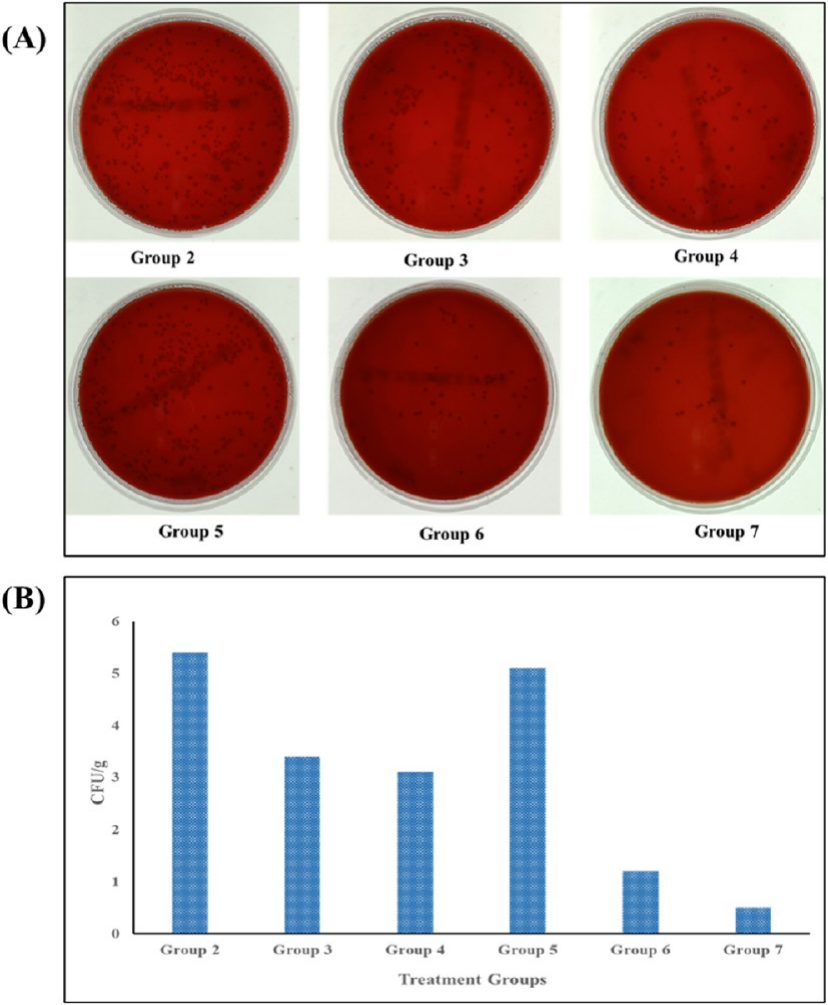
(A) Photographs of *H. pylori* colonies from the treatment groups: (Group 2) Positive control (5.4 CFU/g), (Group 3) Free drug (AMO, MTZ, and PAN) (3.4 CFU/g), (Group 4) Free drug (AMO, MTZ, and PAN) + PIP (3.1 CFU/g), (Group 5) Blank formulation (5.1 CFU/g), (Group 6) M-GRDDS beads of (AMO, MTZ, and PAN) (1.2 CFU/g), and (Group 7) M-GRDDS beads of (AMO, MTZ, PIP, and PAN) (0.5 CFU/g). (B) Bar chart summarizing the CFU/g across the treatment groups, demonstrating the significant reduction of bacterial viability in Groups 6 and 7 treated with the M-GRDDS beads, compared to Group 3 and 4, treated with free drugs.

In comparison to free antibiotics, our investigations indicated that encapsulating drugs in mucoadhesive-GRDDS beads enhanced therapeutic efficiency. This is mainly because the drugs are retained in the stomach for longer, are released sustainably, and are protected from degradation in the stomach’s acidic environment. Similar outcomes were reported by Patil et al., and Sahin et al., where chitosan–alginate mucoadhesive systems showed improved localized delivery and enhanced antimicrobial activity through prolonged gastric residence and targeted drug release.^68,71^ In our formulation, the observed superior performance could be attributed to multiple synergistic mechanisms, such as sustained release and enhanced bioavailability, combined with the adjuvant additive effect of piperine. By improving treatment efficacy and lowering the risk of bacterial resistance and side effects like gastrointestinal irritation that are frequently linked to free drug administration, the fabricated formulation is expected to improve clinical outcomes.

#### 3.4.2 Urease Reaction Test

Production of the urease enzyme by *H. pylori* is a survival adaptation of the organism. Urease produced by the organism helps the production of ammonia, which in turn helps the organism to neutralize and survive the acidic gastric environment. The presence of urease is thus a diagnostic test to confirm the presence of infection. Results of the urease test is provided in Figure 11. Healthy control group 1 (without *H. pylori* infection) did not show any color change, inferring that there was no urease enzyme present in the mucosal tissue, confirming the absence of *H. pylori* infection in the group. Group 2 (positive control) animal infected with *H. pylori* infection showed significant pink color due to the urease reaction, which confirmed the presence of *H. pylori* infection. Compared to the positive control, the color intensity in the treated groups 3, 4, 5, 6, and 7 was lesser, proving the efficacy of treatment. As can be seen in the figure, the color reaction in Group 7 (treated with the GRDDS beads of AMO, MTZ, and PIP along with the enteric-coated beads of PAN) was less prominent and similar to that of a healthy control, proving the eradication efficacy of the prepared GRDDS beads. Urease test confirmed both the presence and eradication of *H. pylori* infection, as indicated by a pH-driven color change. Similar diagnostic applications were highlighted by Costa et al., and Uotani et al., who emphasized the high sensitivity and specificity of the rapid urease test in identifying *H. pylori* infection through enzymatic activity in gastric biopsy specimens.^74,75^ In our study, the diminished color intensity validates the efficacy of localized, sustained drug release from the mucoadhesive beads in targeting and eradicating *H. pylori*.

**Figure 11:**
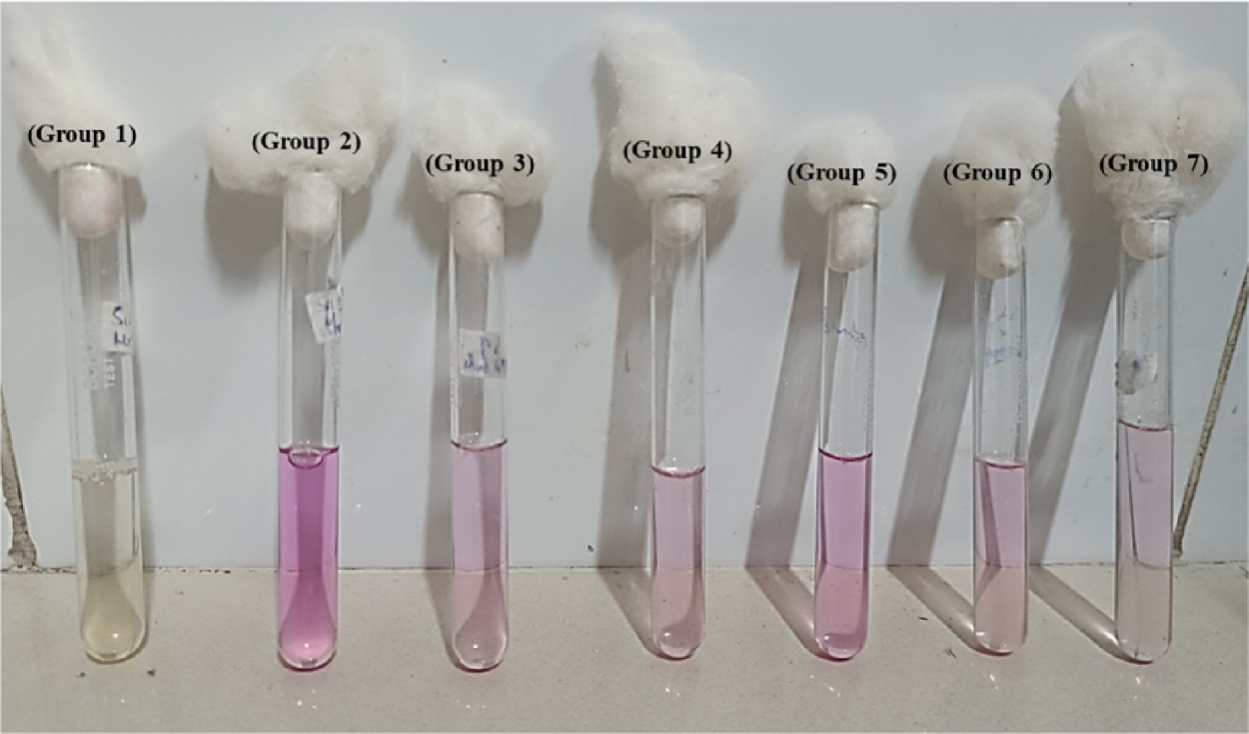
Urease reaction test of different groups: (Group 1) Control, (Group 2) Positive control, (Group 3) Free drug (AMO, MTZ, and PAN), (Group 4) Free drug (AMO, MTZ, and PAN) + PIP, (Group 5) Blank formulation, (Group 6) M-GRDDS beads of (AMO, MTZ, and PAN), and (Group 7) M-GRDDS beads of (AMO, MTZ, PIP, and PAN).

#### 3.4.3 Interleukin-1 Beta (IL-1β) Levels

The powerful pro-inflammatory cytokine IL-1β is markedly elevated during *H. pylori* infection. Along with decreasing parietal cell function, it causes achlorhydria, or decreased stomach acid output, which worsens the condition by fostering an environment that promotes tissue damage and bacterial persistence. The results from our study Figure 12A demonstrate how the absence of inflammatory stimuli resulted in significantly lower IL-1β levels in the healthy control group compared to the positive control group (*H. pylori*-infected, untreated). Reduced IL-1β levels were seen in the groups treated with free drugs (AMO, MTZ, and PAN), and the level was further reduced when these drugs were given with PIP. This indicates that these treatments decrease inflammation, with PIP possibly boosting drug efficacy by increasing bioavailability or because of its inherent anti-inflammatory effects. IL-1β levels were not significantly affected by the blank formulation group (placebo beads), showing values similar to the positive control, suggesting that the carrier alone did not have any therapeutic effect. The drug loaded mucoadhesive-GRDDS beads of AMO, MTZ, and enteric-coated PAN demonstrated a better inhibition of IL-1β levels as compared to the positive control and other groups, demonstrating better drug delivery and localized anti-inflammatory effects resulting from the gastroretentive drug delivery. The statistical evaluation using the Student’s *t* test showed that the enhancement in the activity showed by the formulation was significant (*p* ≤ 0.01). As seen in the case of free PIP, the mucoadhesive formulation of PIP also resulted in an additive action. The improved efficacy could be attributed to the better drug penetration and control of cytokine production, resulting in anti-inflammatory action. The results demonstrated significantly elevated IL-1β levels in *H. pylori*-infected groups compared to healthy controls, consistent with the established role of IL-1β; as a key pro-inflammatory cytokine driving gastric inflammation and contributing to carcinogenesis. Similar findings have been reported by Yoo et al., and Lamb and Chen, who described how *H. pylori* infection activates NF-κB signaling pathways that upregulate IL-1β expression and promote gastric pathology.^76,77^ In our study, treatment with free drug combinations reduced IL-1β expression, while the addition of PIP further enhanced this anti-inflammatory effect due to its bioavailability-enhancing and intrinsic modulatory properties. Importantly, the drug loaded mucoadhesive-GRDDS beads produced the greatest suppression of IL-1β, reflecting the benefits of localized drug delivery, deeper mucosal penetration, and sustained release kinetics in controlling chronic gastric inflammation. These findings corroborate the therapeutic value of drug loaded M-GRDDS systems in reducing *H. pylori* induced inflammation and mitigating long-term gastric complications.

**Figure 12:**
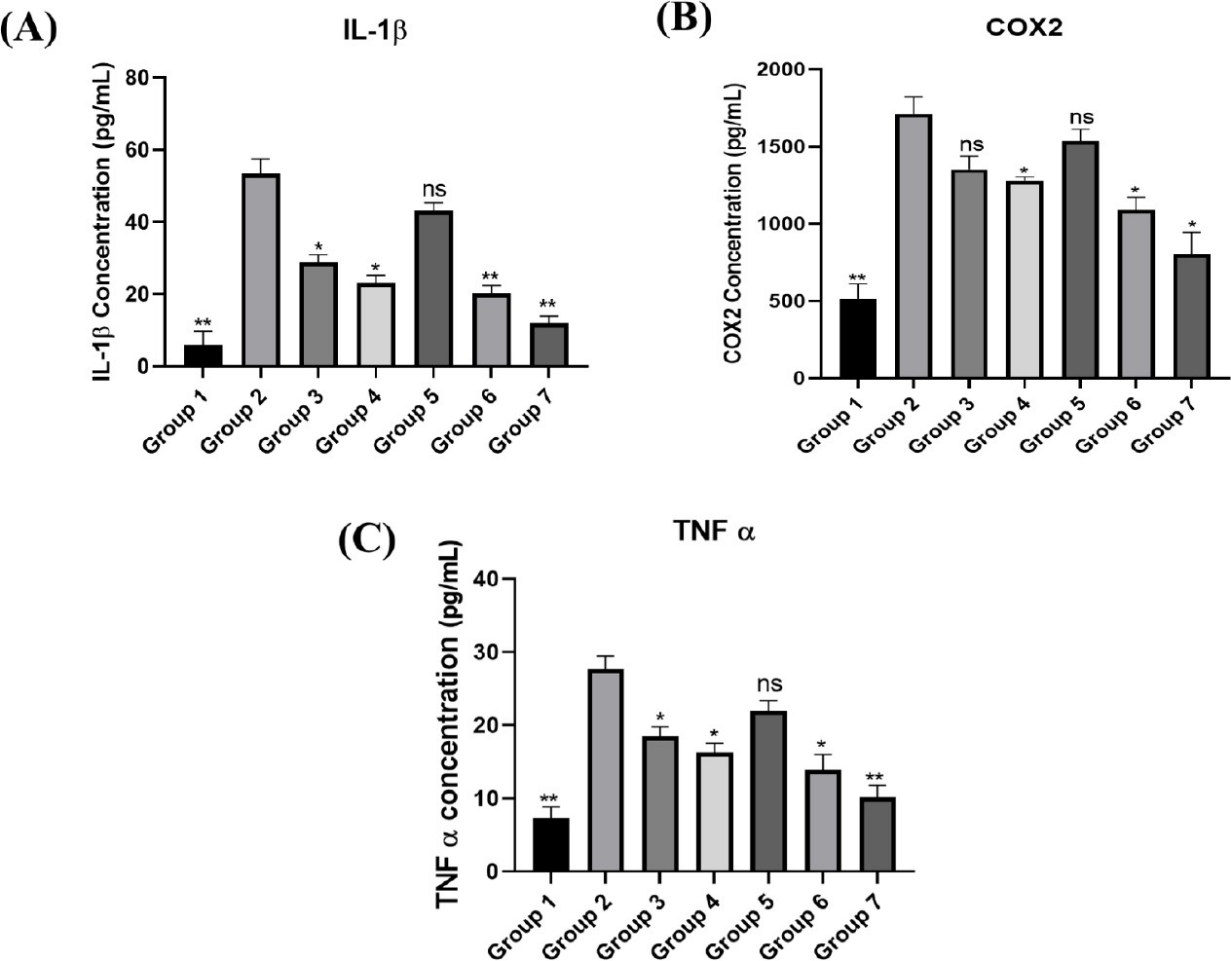
Bar graphs depicting relative expressions of (A) IL-1β, (B) COX-2, and (C) TNF-α compared with sham control. *p < 0.05, **p < 0.01.

#### 3.4.4 Cyclooxygenase-2 (COX-2) Levels

COX-2 becomes activated and elevated in response to inflammatory stimuli. The virulence factor VacA of *H. pylori* is associated with COX-2 overexpression in gastric ulcers. In addition, COX-2 overexpression is linked to the development and dissemination of gastric cancer. The results from our study depicted as bar chart (Figure 12B) demonstrate how the absence of inflammatory stimuli resulted in significantly lower COX-2 levels in the healthy control group compared to the positive control group (*H. pylori*-infected, untreated). Reduced COX-2 levels were seen in the groups treated with free drugs (AMO, MTZ, and PAN), and the levels were further reduced when these drugs were given with PIP. This indicates that these treatments decrease inflammation, while the inclusion of PIP found to further enhace drug efficacy by increasing bioavailability or because of its inherent anti-inflammatory effects. COX-2 levels in the gastric tissue were not significantly affected by the blank formulation group (placebo beads), showing values similar to the positive control. This indicates that the blank formulation alone does not have any therapeutic value and does not affect inflammation. The mucoadhesive-GRDDS beads of AMO, MTZ, and enteric-coated PAN demonstrated a better inhibition of COX-2 levels as compared to the positive control and other groups, demonstrating better drug delivery and localized anti-inflammatory effects resulting from the gastroretentive drug delivery. The statistical evaluation using the Student’s *t* test showed that the enhancement in the activity indicated by the formulation was significant (*p* ≤ 0.05). As seen in the case of free PIP, the mucoadhesive formulation of PIP also resulted in an additive action. The improved efficacy could be attributed to the better drug penetration and control of cytokine production, resulting in anti-inflammatory action. The results showed significantly elevated COX-2 levels in the *H. pylori*-infected group compared to healthy controls, consistent with established evidence that *H. pylori* induces COX-2 overexpression via pro-inflammatory signaling pathways influenced by virulence factors such as VacA. Similar observations were reported by Shao et al., and Cheng and Fan, who highlighted COX-2 as a pivotal mediator in *H. pylori*-associated gastric inflammation and carcinogenesis.^78,79^ In our study, treatment with free drugs reduced COX-2 levels, with further suppression achieved when combined with PIP, attributable to enhanced drug bioavailability and PIP’s intrinsic anti-inflammatory effects. The mucoadhesive GRDDS beads exhibited the most pronounced inhibition of COX-2, demonstrating the advantage of localized drug delivery and sustained release in controlling gastric inflammation. These findings reinforce the therapeutic value of GRDDS beads in improving eradication efficiency, reducing inflammation-associated gastric complications, and lowering long-term cancer risk.

#### 3.4.5 Tumor Necrosis Factor-α (TNF-α) Levels

TNF-α is the main cytokine in the inflammatory cascade, leading to gastric ulcer and cancer. *H. pylori* triggers the generation of TNF-α from immune cells (such as neutrophils and macrophages) and stomach epithelial cells, increasing the inflammatory responses. To aggravate mucosal injury, TNF-α stimulates nuclear factor-kappa β (NF-kβ), a transcription factor that upregulates other pro-inflammatory mediators such as IL-1β and IL-6. The results from our study (Figure 12C) demonstrate how the absence of inflammatory stimuli resulted in significantly lower TNF-α levels in the healthy control group (Group 1) compared to the positive control group (*H. pylori*-infected, untreated- Group 2). Reduced TNF-α levels were seen in the groups treated with free drugs (AMO, MTZ, and PAN), and the level was further reduced when these drugs were given with PIP. This indicates that these treatments decrease inflammation, with PIP possibly boosting drug efficacy by increasing bioavailability or because of its inherent anti-inflammatory effects. TNF-α levels were not significantly affected by the blank formulation group (Group 5), showing values similar to the positive control, suggesting that the carrier alone had no therapeutic effect. The mucoadhesive GRDDS beads of AMO, MTZ, and enteric-coated PAN demonstrated a better inhibition of TNF-α levels as compared to the positive control and other groups, demonstrating better drug delivery and localized anti-inflammatory effects resulting from the gastroretentive drug delivery. The Student’s *t*-test statistical evaluation showed that the formulation’s activity enhancement was significant (*p* ≤ 0.01). As seen in the case of free PIP, the mucoadhesive formulation of PIP also resulted in an additive action. The improved efficacy could be attributed to better drug penetration and control of cytokine production, resulting in anti-inflammatory action. Results underscore the critical role of TNF-α in *H. pylori*-induced gastric inflammation as reported by Morningstar-Wright et al., Bravo et al.^80,81^

#### 3.4.6 Gene Expression

Results of gene expression studies depicted in Figure 13A show that the level of TNF-α expression is nearly 20 times higher the disease control group, which was infected with *H. pylori* but not treated, than in the healthy controls. This agrees with the strong inflammatory responses that would be expected from a protracted bacterial presence. The TNF-α expression was only 14 times higher in the group treated with the free drug (AMO, MTZ, and PAN) and only 11 times in the group treated with free drugs along with PIP when the expression was compared with the healthy control. The mucoadhesive-GRDDS beads of AMO, MTZ, and enteric-coated PAN demonstrated a better inhibition of TNF-α expression, which was only 7 times as compared to the healthy control group, demonstrating better drug delivery of the GRDDS system. The statistical evaluation using the Student’s *t*-test showed that the enhancement in the activity shown by the formulation was significant. As seen in the case of free PIP, the mucoadhesive formulation of PIP also resulted in an additive action. The improved efficacy could be attributed to the better drug penetration and control of the TNF-α expression, resulting in anti-inflammatory action.

**Figure 13:**
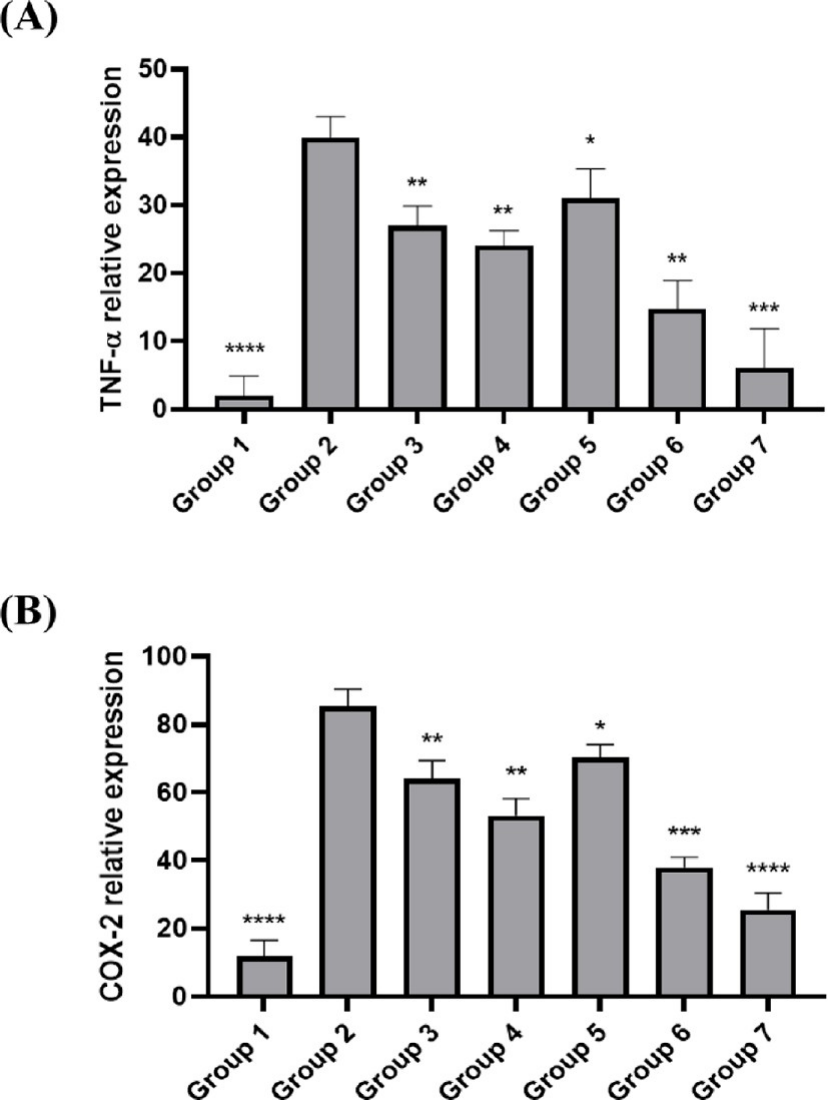
Bar graphs depicting relative gene expression of (A) TNF-α and (B) COX-2 compared with sham control. *p < 0.05, **p < 0.01, ***p < 0.001, ****p < 0.0001.

COX-2 expression depicted in Figure 13B shows a 7 times higher expression in the disease control group infected with *H. pylori* but not treated, than in the healthy controls. This could be attributed to the strong inflammatory responses that would be expected from a prolonged bacterial presence. The COX-2 expression was only 5 times higher in the group treated with the free drug (AMO, MTZ, and PAN) and only 4 times in the group treated with free drugs along with PIP when the expression was compared with the healthy control. The mucoadhesive-GRDDS beads of AMO, MTZ, and enteric-coated PAN demonstrated a better inhibition of COX-2 expression, which was only 3 times as compared to the healthy control and other groups, demonstrating better drug delivery of the GRDDS system. The statistical evaluation using the Student’s *t* test showed significant enhancement in the activity shown by the formulation. As seen in the case of free PIP, the mucoadhesive formulation of PIP also resulted in an additive action. These findings are consistent with reports by Morningstar-Wright et al., Rahimian et al., and Thalmaier et al., who demonstrated that *H. pylori*-induced TNFα and COX-2 upregulation drive chronic gastric inflammation, and that targeted interventions mitigating the expression of these cytokines can significantly reduce mucosal damage.^80,82,83^ Collectively, our results emphasize the therapeutic potential of M-GRDDS formulation in modulating key pro-inflammatory mediators to improve treatment outcomes for *H. pylori* infection.

#### 3.4.7 Results of Histopathology

Histopathology data is displayed in Figure 14. A healthy tissue architecture was seen with normal stomach mucosa in the healthy control group, which had intact epithelial layers (labeled as black arrow), well-organized gastric glandular structures (labeled as star), and no infiltration of inflammatory cells in the lamina propria. The *H. pylori*-infected, untreated disease control group, on the other hand, showed signs of severe ulceration, involving profound erosions of the epithelium, extensive destruction of granular structures, and neutrophils (red arrow) in the mucosa and submucosa. With an ulcer index value of 3 (severe), the ulcerated sites showed focal areas of necrosis (circle), significant edema, and bleeding. The group treated with free drugs (AMO, MTZ, and PAN) demonstrated a modest improvement, with a decreased ulcer depth and a partially repaired epithelial layer, although significant inflammation persisted with major neutrophil infiltration and a few damaged gastric glands (inflammation score:2). Although some erosions were noticed, the inclusion of PIP to free medicines slightly accelerated the healing process by demonstrating reduced inflammatory infiltration and enhanced glandular organization (inflammation score: 1.5). The drug free placebo bead demonstrated no evidence of tissue healing (inflammation score: 2.5) and maintained ulceration and inflammation equivalent to disease control. On the other hand, the group that was treated with the drug loaded mucoadhesive-GRDDS beads of AMO and MTZ showed significant healing with low edema (inflammation score: 1), the mucosa demonstrated significant re-epithelialization, decreased inflammatory cell infiltration (primary moderate lymphocytic presence), and restored glandular architecture. The group that received enteric-coated PAN beads and mucoadhesive GRDDS beads loaded with AMO, MTZ, and PIP showed the most significant improvement, similar to that seen with the healthy control group (inflammation score: 0.5). This group showed only limited inflammatory cells in the lamina propria (red arrow), intact glandular structures, and almost complete epithelial regeneration. Effective tissue healing was achieved as evidenced by the low level of fibrosis and the presence of angiogenesis. These results demonstrate the effectiveness of the mucoadhesive-GRDDS bead formulation, mainly when used together with PIP, in aiding mucosal regeneration. These results are consistent with the documented histopathological features of *H. pylori* infection described by Wang et al., and the evidence from Kong et al., that effective eradication and controlled delivery systems markedly improve gastric mucosal recovery.^84,85^ Our findings demonstrate that the developed mucoadhesive GRDDS not only eradicates *H. pylori* but also supports mucosal healing and tissue regeneration, essential for long-term gastric health.

**Figure 14:**
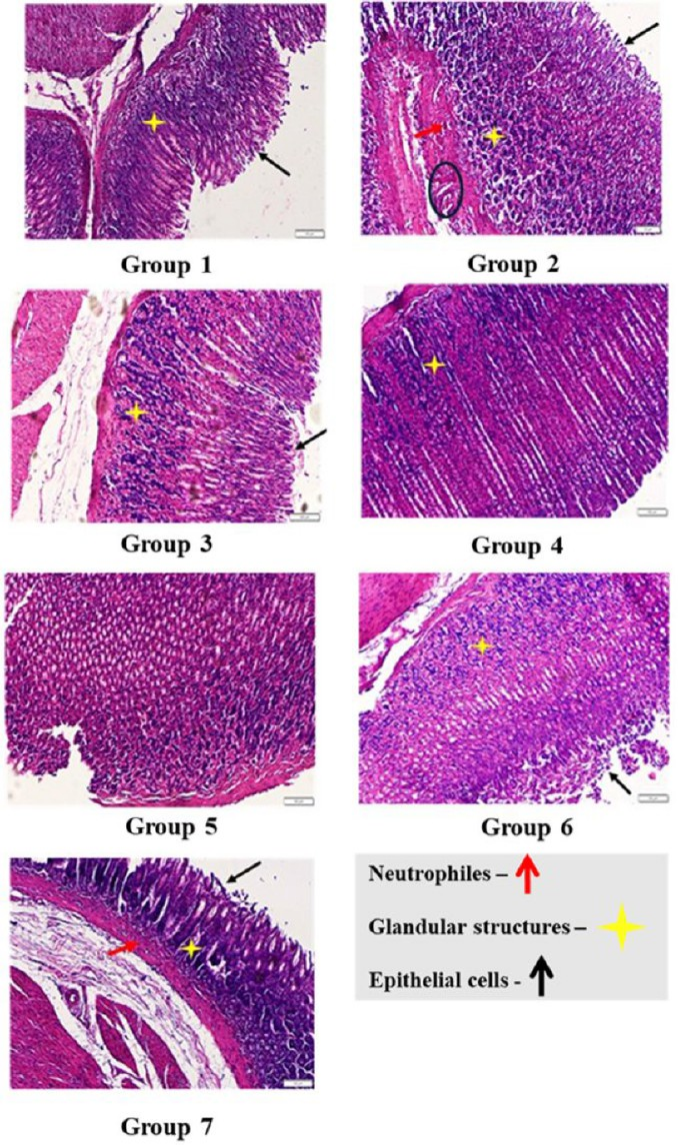
Histopathology images of the treated animal model with (Group 1) Control, (Group 2) Positive control, (Group 3) Free drug (AMO, MTZ, and PAN), (Group 4) Free drug (AMO, MTZ, and PAN) + PIP, (Group 5) Blank formulation, (Group 6) M-GRDDS beads of (AMO, MTZ, and PAN), and (Group 7) M-GRDDS beads of (AMO, MTZ, PIP, and PAN).

### 3.5 Stability of Drug-Loaded M-GRDDS Beads at Intermediate and Accelerated Conditions

To determine the integrity of the encapsulated drugs over time, the prepared GRDDS beads were exposed to the intermediate and accelerated stability conditions as per the ICH guidelines. The results obtained in terms of the % degradation are displayed in Figure 15. The % deterioration observed was less than 10% indicating that the formulation is highly stable on storage. This is important in predicting shelf life and the effectiveness of therapies. The drug concentration observed in the formulation on storage at 30 ± 2 °C/65 ± 5% RH (intermediate conditions) after 6 months were AMO 93.64 ± 1.04%, MTZ 92.37 ± 1.49%, PAN 92.74 ± 1.22% and PIP 93.03 ± 1.46%. The concentration observed at 40 ± 2 °C/75 ± 5% RH (accelerated conditions) after 6 months of storage was AMO 91.37 ± 1.08%, MTZ 92.32 ± 1.23%, PAN 92.69 ± 1.95% and PIP 92.93 ± 1.24%. These percentages show how much of the original drug content was left after storage, demonstrating how well the beads preserved the drugs from environmental effects. As per the ICH guidelines, the observed % degradation was well within the acceptable limits of not more than 10%. The results corroborate the protective nature of the GRDDS system from the moisture and heat stress, assuring long-lasting effectiveness for the drugs in managing *H. pylori*. The stability study results demonstrate that the GRDDS mucoadhesive beads maintain drug integrity well under both intermediate (30 ± 2 °C/65 ± 5% RH) and accelerated (40 ± 2 °C/75 ± 5% RH) ICH storage conditions over 6 months, with less than 10% drug degradation observed for AMO, MTZ, PAN, and PIP. This level of stability is crucial for ensuring the shelf life, effectiveness, and consistent therapeutic performance of the GRDDS system in managing *H. pylori* infections, which is in compliance with regulatory expectations for pharmaceutical products.^52,86^

**Figure 15:**
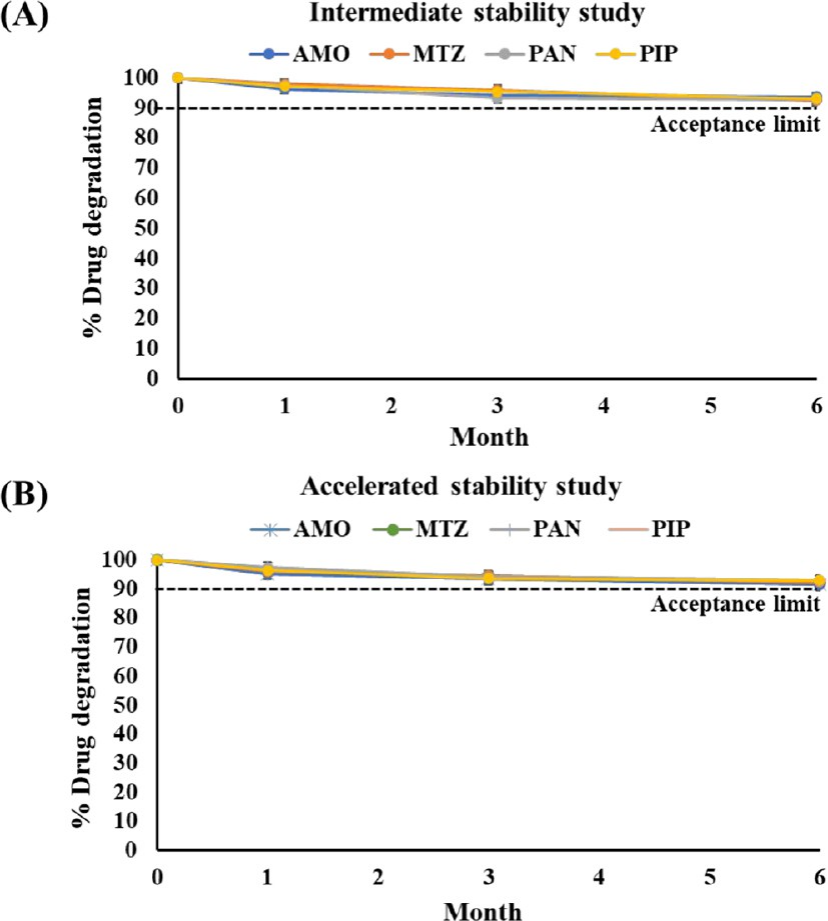
Stability study of the prepared beads; (A) intermediate stability condition, (b) accelerated stability condition.

## 4 Conclusion

A novel mucoadhesive GRDDS system to orally deliver AMO, MTZ, PAN, and PIP for the eradication of *H. pylori* infection has been developed. The optimized bead size and density facilitated the controlled release as evidenced in the *in vitro* release study results, which was in tandem with the prolonged gastric retention observed in the *in vivo* rabbit model. The mucoadhesive strength measured through the wash-off method correlated well with the *in vivo* mucosal adherence, supporting the formulation’s ability to remain localized at the gastric site. In the rabbit model, the formulation showed a mucoadhesion of up to 8 h. The *in vitro* release showed that the alginate beads coated with chitosan successfully delivered drugs in a sustained manner, providing extended therapeutic efficacy. Results of the *in vivo* gastric mucosal bioabvailability study corelated well with the results of *in vitro* drug release study confirming that the drug loaded mucoadhesive-GRDDS beads facilitated localized drug bioavailability at the gastric mucosal surface. The concentration of AMO, MTZ, and PIP in the stomach mucosa was noticeably higher in the group treated with mucoadhesive beads than in the group treated with free drugs of the same. The *in vivo* efficacy evaluation of the prepared GRDDS beads showed better eradication of bacteria than the free drug. A significant decrease in the CFU count (*p* < 0.05 in comparison to the positive control) indicated the higher efficiency of the formulation. The biochemical and gene expression tests also showed the treatment efficacy of the drug loaded M-GRDDS system for treating *H. pylori*-based gastric ulcers. The histopathology evaluation demonstrated the effectiveness of the mucoadhesive bead formulation, especially when given together with PIP in promoting mucosal regeneration. The intermediate and accelerated stability study revealed that the GRDDS beads could protect the drugs from degradation during storage under various elevated temperature and humidity conditions, assuring long-lasting effectiveness in managing *H. pylori*. These observations conclude that the developed GRDDS formulation holds significant potential for localized drug action and improves bioavailability, contributing to more effective eradication of *H. pylori*-based gastric ulcer.

## 5 Limitation and Future Work

A limitation of the GRDDS mucoadhesive beads is the possibility of prolonged gastric retention of beads, which may pose risks such as gastric irritation, nausea, bloating, delayed gastric emptying, or, in rare cases, gastric obstruction, particularly when the dosage form is large or improperly administered. To mitigate these concerns, the beads must be formulated using biocompatible and biodegradable polymers that gradually disintegrate, ensuring controlled drug release and minimizing the potential for accumulation in the stomach. Additionally, bead size and density should also be carefully optimized to achieve a balance between effective gastric retention and safe transit.

Future studies warrant clinical evaluations to be carried out to evaluate the effectiveness of the formulation in eradicating the *H. pylori* infection and related peptic ulcer disease (PUD) in humans. Safety evaluations to monitor gastrointestinal side effects through comprehensive clinical assessments, imaging techniques, and patient-reported outcomes. Measures such as dose titration, evaluation of food effects, and exclusion of individuals with known gastric motility disorders will further help reduce associated risks. These precautions and adherence to ethical and regulatory standards will support the safe evaluation of the gastroretentive system’s therapeutic potential.

## Supplementary Material



## Notes

Additional experimental results: Supplementary Tables 1, 2, and 3 shows the formulation optimization trials for fabrication of mucoadhesive beads of AMO, MTZ, and PIP; Supplementary Table 4 shows the formulation optimization trials for fabrication of enteric coated bead of PAN; Supplementary Figure 1. depicts the physical appearance of prepared mucoadhesive beads (PDF)
