# Supplementary material for: Design and Characterization of Novel Gastroretentive Drug Delivery System of Antibiotics and Piperine for the Eradication of H. pylori Infection
Source: Mol Pharm. 2025 Nov 14;22(12):7641–63. doi: 10.1021/acs.molpharmaceut.5c01253 (PMC12673577; doi:10.1021/acs.molpharmaceut.5c01253)
Supplement: Supplementary file 1 [file mp5c01253_si_001.pdf]

Supporting Information

Design and Characterization of Novel Gastroretentive Drug Delivery System of Antibiotics and Piperine for the Eradication of *H. pylori* Infection

Ashutosh Gupta[1], Moumita Saha[1], Shivani Shailesh Kunkalienkar[1], Aadarsh Ghurye[2], Shweta Verma[3], Jahnavy Joshi[3], Abhishek Jha[4], Srinivas Mutalik[5], Shiran Shetty[6], Raghu Chandrashekar Hariharapura[2], Ashwini Aithal[7], K Nandakumar[8], Raviraja N. Seetharam[3], Sudheer Moorkoth[1*]

1. Department of Pharmaceutical Quality Assurance, Manipal College of Pharmaceutical Sciences, Manipal Academy of Higher Education, Manipal 576104, Karnataka, India
2. Department of Pharmaceutical Biotechnology, Manipal College of Pharmaceutical Sciences, Manipal Academy of Higher Education, Manipal 576104, Karnataka, India
3. Manipal Centre for Biotherapeutics Research, Manipal Academy of Higher Education, Manipal 576104, Karnataka, India
4. Department of Pharmaceutics, Dr. D. Y. Patil Institute of Pharmaceutical Sciences and Research Pimpri, Pune-18, India
5. Department of Pharmaceutics, Manipal College of Pharmaceutical Sciences, Manipal Academy of Higher Education, Manipal 576104, Karnataka, India.
6. Department of Gastroenterology and Hepatology, Kasturba Medical College, Manipal Academy of Higher Education, Manipal 576104, Karnataka, India
7. Division of Anatomy, Department of Basic Medical Sciences, Manipal Academy of Higher Education, Manipal 576104, Karnataka, India
8. Department of Pharmacology, Manipal College of Pharmaceutical Sciences, Manipal Academy of Higher Education, Manipal 576104, Karnataka, India

***Corresponding author**

Sudheer Moorkoth

Department of Pharmaceutical Quality Assurance, Manipal College of Pharmaceutical Sciences, Manipal Academy of Higher Education, Manipal 576104, Karnataka, India

Email address: moorkoth.s@manipal.edu

Supplementary data

Table

Supplementary Table 1. Formulation optimization trials for fabrication of mucoadhesive beads of AMO

**Sodium alginate (%)**	**Chitosan (%)**	**Calcium chloride (%)**	**Drug added (mg)**	**Entrapment efficiency %**
**3**	0.75	4	100	14.87
**4**	0.75	8	100	21.39
**5**	0.75	6	100	38.81
**5**	0.5	10	100	45.34
**7**	0.5	10	100	51.33
**9**	0.5	9	100	62.18
**9**	**0.5**	**10**	100	**84.71**

Supplementary Table 2. Formulation optimization trials for fabrication of mucoadhesive beads of MTZ

Sodium alginate (%)	Chitosan (%)	Calcium chloride (%)	Drug (mg)	Entrapment efficiency %
**4**	0.5	10	100	22.79
**5**	0.5	10	100	37.97
**6**	0.5	10	100	44.87
**7**	0.5	10	100	59.47
**8**	0.5	10	100	74.23
**9**	**0.5**	**10**	**100**	**82.61**

Supplementary Table 3. Formulation optimization trials for fabrication of mucoadhesive beads of PIP

**Sodium alginate (%)**	**Chitosan (%)**	**Calcium chloride (%)**	**Drug (mg)**	**Entrapment efficiency %**
**5**	0.5	10	100	38
**6**	0.5	10	100	49
**7**	0.5	10	100	58
**8**	0.5	10	100	67
**8**	0.5	10	100	79
**9**	**0.5**	**10**	**100**	**86**

Supplementary Table 4. Formulation optimization trials for fabrication of mucoadhesive beads of PAN

**Sodium alginate (%)**	**Chitosan (%)**	**Calcium chloride (%)**	**Drug (mg)**	**Entrapment efficiency %**
**6**	0.5	10	100	57
**7**	0.5	10	100	71
**8**	**0.5**	**10**	**100**	**85**

*For the enteric coating of the prepared pantoprazole-loaded beads, 0.4% Eudragit L 30 D-55 polymer was used.

Figure

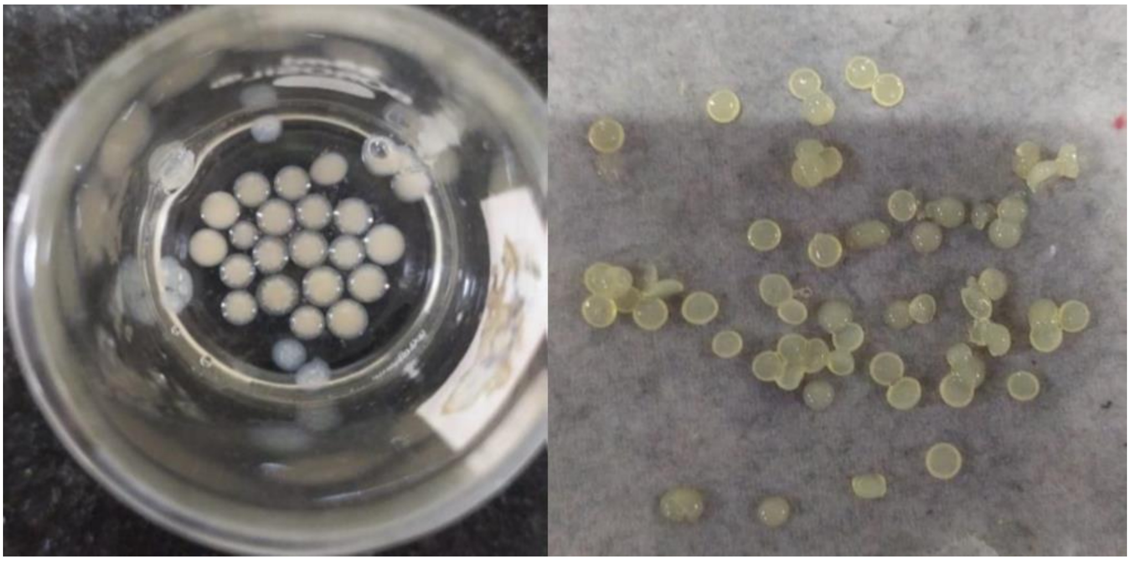

Supplementary Fig. 1. The physical appearance of prepared mucoadhesive beads
